# Novel pre-clinical mouse models for chronic Graft-versus-Host Disease

**DOI:** 10.3389/fimmu.2022.1079921

**Published:** 2023-01-24

**Authors:** Lydia Verlaat, Katarina Riesner, Martina Kalupa, Beate Jung, Sarah Mertlitz, Constanze Schwarz, Jörg Mengwasser, Claudine Fricke, Olaf Penack

**Affiliations:** Department of Hematology, Oncology and Tumorimmunology, Charité – Universitätsmedizin Berlin, Corporate Member of Freie Universität Berlin and Humboldt-Universität zu Berlin, Berlin, Germany

**Keywords:** allogeneic hematopoietic stem cell transplantation, chronic Graft-versus-Host Disease, mouse models, G-CSF, xenogeneic transplantation, fibrosis, inflammation

## Abstract

Despite considerable progress in allogeneic hematopoietic cell transplantation (allo-HCT) has been achieved over the past years, chronic Graft-versus-Host Disease (cGvHD) still contributes to high morbidity rates, thus remaining a major hurdle in allo-HCT patients. To understand the complex pathophysiology of cGvHD and to develop refined prophylaxis and treatment strategies, improved pre-clinical models are needed. In this study, we developed two murine cGvHD models, which display high long-term morbidity but low mortality and depict the heterogeneous clinical manifestations of cGvHD seen in patients. We established a haploidentical C57BL/6→B6D2F1 allo-HCT model that uses myeloablative radiation and G-CSF-mobilized splenocytes as stem cell source and a sub-lethally irradiated Xenograft model, which utilizes the transfer of human peripheral blood mononuclear cells (PBMCs) into NOD scid gamma (NSG)-recipients. We characterized both mouse models to exhibit diverse clinical and histopathological signs of human cGvHD as extensive tissue damage, fibrosis/sclerosis, inflammation and B cell infiltration in cGvHD target organs skin, liver, lung and colon and found a decelerated immune cell reconstitution in the late phase after HCT. Our pre-clinical models can help to gain a deeper understanding of the target structures and mechanisms of cGvHD pathology and may enable a more reliable translation of experimental findings into the human setting of allo-HCT.

## Introduction

Allogeneic hematopoetic cell transplantation (allo-HCT) has emerged to the therapy of choice for a variety of hematologic, congenital and neoplastic disorders. Until 2020, nearly 50,000 allo-HCTs have been performed worldwide every year (1) with increasing numbers since 1990 (2). Although treatment outcomes have significantly improved, chronic Graft-versus-Host Disease (cGvHD) still occurs in up to 70% of transplanted patients and is accompanied with 25% of deaths after allo-HCT (3). Thus, cGvHD remains one of the major allo-HCT sequelae and contributes substantially to high long-term morbidity and non-relapse related mortality (NRM) rates (4, 5).

Due to the rising age of patients or unrelated-donor- and MHC-mismatched-transplantations, the overall incidence of cGvHD is increasing recently (6). Other major risk factors include prior acute GvHD and peripheral blood (PB) as hematopoietic cell (HC) source (7). Since HC collection from PB is easier to conduct, less invasive and bears a decreased risk for complications, it has recently become the standard choice in allo-HCT settings. Donors get administered with hematopoetic growth factors prior to apheresis, usually granulocyte colony-stimulating factor (G-CSF), which mobilizes bone marrow (BM) resident HCs into the PB circulation (8). However, several studies propose an increased risk of cGVHD development after G-CSF–mobilized allo-HCT (9, 10).

The pathophysiology of cGvHD development is highly complex (11) and still not fully understood. The establishment of mouse models has been of central importance for the identification and the general understanding of the cGvHD pathophysiology and the pre-clinical testing of reliable treatment strategies for translation into humans. Of big advantage, the genetically determined MHC variability between donors and recipients can be tightly controlled in murine models (12–14). For the majority of models, inbred mouse strain recipients are conditioned with total body irradiation and subsequently transplanted with HC grafts from BM, which is supplemented with T cells from donor mice (15). Hence, the severity and onset of GvHD can be adjusted by adaption of radiation doses and T cell numbers (16).

As reviewed elsewhere (15, 17, 18) several distinct mouse models were established, which helped to understand the intricate cGvHD pathophysiology and facilitated interventional treatment strategies. cGvHD mouse models can be broadly categorized into sclerodermatous/pro-fibrotic models, which exhibited fibrotic depositions in skin, lung or liver within 30 days after transplantation (19–21), autoantibody-mediated/lupus-like models that resemble human cGvHD, as they showed lupus-like manifestations as nephritis, liver cirrhosis, salivary gland fibrosis and skin involvement (22–24) and a transgenic thymic dysfunction model (25). Some mouse models manage to mimic a mixed cGvHD phenotype combining autoantibody production and scleroderma (26) or progressing from acute to chronic GvHD (27). Since this overlapping transformation is common in patients, mouse models can help to identify mechanisms of the causal link between allo- and autoreactivity.

Beyond classical murine models, xenogeneic (humanized) models have been developed, where human peripheral blood mononuclear cells (PBMCs) can be transplanted into immunodeficient mice. As one example, NOD/SCID gamma (NSG) mice are deficient in their Interleukin 2-receptor-function, resulting in a missing T-, B- and Natural Killer (NK) cell repertoire and deficient macrophage- and dendritic cell (DC) activity (28). In xeno-HCT experiments this model showed promising engraftment numbers and an acute GvHD phenotype with human T-cell infiltration of mouse skin, liver, intestine and lungs (29). Although this setup could be used to study and modify human GvHD *in vivo*, humanized models for simulation of cGvHD have not been further described.

Nonetheless, there is still no individual mouse model reliably comprising the highly diverse pathological and immunological characteristics of cGvHD seen in humans. So far, researchers may use more than one model to study cGvHD depending on ascientific hypothesis.Klicken Sie hier, um Text einzugeben.Furthermore, nearly all murine models are transplanted with HCs generated from BM, whereas the majority of clinical allo-HCTs are performed using G-CSF mobilized donor PB cells due to the simplified cell-collection procedure.

### Objective

The present study attempts to establish pre-clinically relevant murine cGvHD models, which display high long-term morbidity but low mortality and resemble diverse manifestations of cGvHD seen in patients. The murine parent-into F1 model C57BL/6→B6D2F1 uses G-CSF-mobilized splenocytes as stem cell source, while the humanized model applies transplantation of human PBMCs → NSG mice. Those novel models can help to understand and describe the cGvHD pathology and to develop alternative therapeutic strategies in cGvHD prevention and management and guarantee a more reliable translation of the experimental findings into the human setting of allo-HCT.

## Materials and methods

### Mice

Female C57BL/6NCrl (B6) (H-2K^b^) and B6D2F1 (BDF1) (H-2K^b/d^) mice were purchased from Charles River Laboratories (Sulzfeld, Germany). Female immunodeficient NOD scid gamma (NSG) (NOD.Cg-Prkdc^scid^ Il2rg^tm1Wjl^/SzJ) were bred and ordered from the group of Prof. Hans-Dieter Volk (Charité/BIH, Berlin, Germany). All animals were housed in a 12-hour light-dark cycle under specified pathogen-free conditions in the Charité Animal Facility (Forschungseinrichtung für experimentelle Medizin). All experiments were approved by the local Ethics Committee for Animal Research (State Office of Health and Social Affairs, LaGeSo) and executed in compliance with the European Union guidelines.

### Transplantation

Donor mice were aged >20 weeks, recipients were aged at least 8 to 10 weeks before HCT. B6 and BDF1 donors received daily subcutaneous injections of 10 mg/kg G-CSF (Filgrastim, 48 Mio. U.; Hexal, Germany) in 100 μl of sterile 5% glucose solution (Sigma-Aldrich, USA) for 5 consecutive days prior to transplantation to mobilize HCs. BDF1 underwent 1100 cGy total body irradiation on transplantation-day, split into two doses with a resting phase of at least 4 hours to reduce gut toxicity. NSGs were conditioned with reduced irradiation of 200 cGy on day -1 before HCT. On day 0, whole splenocytes were isolated from B6 donors (alternatively from BDF1-donors as syngeneic (syn) control group) and injected intravenously (i.v.) into conditioned BDF1 recipients. NSG mice were injected i.v. with human PBMCs, isolated from buffy coats from unknown donors provided by the German Red Cross, Berlin. Control NSG mice received only irradiation without injection of human PBMCs.

### GvHD monitoring

cGvHD and control group animals were handled blinded to minimize experimental bias. HCT recipients were individually scored for four clinical parameters (posture, activity, fur, skin) on a scale from 0 to 2. Clinical GvHD score was assessed by summation of all parameters. Animals were sacrificed when exceeding a total GvHD score of 5 or a single GvHD score of >2. Weight loss was scored according to the ‘body condition score’ described by Ullman-Cullere (30) and animals were sacrificed when body condition score exceeded stadium (BC1 ≥ 20% total body weight loss)Survival was monitored daily. cGvHD experiments were run for 125 days before finalization.

### Histological analyzes

Mice were euthanized and Tissue-Tek^®^ cryo-embedding medium (Sakura Finetek, Alphen aan den Rijn, The Netherlands) was injected into the trachea to fill the lungs. Skin, liver, lung and colon were dissected and frozen in -80°C pre-cooled methylbutan (Carl Roth, Germany). 7 µm tissue cryosections were cut with a cryostat, fixed for 30 minutes in 10% ROTI^®^Histofix (Carl Roth, Germany) before Masson’s Trichrome fibrosis staining was performed. Histopathologic and fibrotic grading of GvHD in murine tissue was performed blinded regarding cGvHD and control group in the stained sections after adapted Lerner citeria (31), as described with Cooke et al. (32) and Shulman et al. (33).

For immunohistofluorescence analyzes, 7 µm cryosections were fixed in -20°C methanol (Carl Roth, Germany), blocked (PBS + 3% BSA + 5% FCS) and incubated with primary antibodies (hamster CD3 (145-2C11; 1:200;BD Biosciences, USA), rat-B220 (6D5; 1:200; Biolegend, USA), hamster CD31 (2H8; 1:300; Invitrogen, USA) and rat-Endomucin (V.7C7; 1:200; Santa Cruz Biotech, USA) over night at 4°C. For visualization, sections were counter-stained with secondary antibodies donkey-anti-rat-AF488 (1:500; Invitrogen, USA) and goat-anti-hamster-Cy3 (1:1000; Jackson Laboratories, USA) for 2 hours at room temperature. Nuclei were stained with 2mg/ml 4’,6-Diamidino-2-phenylindole (DAPI; Sigma-Aldrich, USA) before mounting with Fluoromount-G^®^ (Southern Biotech, USA). Microscopy was performed using a Zeiss PALM MicroBeam with X-Cite^®^ Xylis LED light source and Axiocam 305 color/Axiocam 712 mono cameras. Systems were using the ZEN blue software Version 3.1. Whole slide images were acquired from stitched tiles and processed and analyzed in FIJI Version 1.52p (34).

### Flow cytometry

PB of mice was collected by facial vein or tail vein puncture. For analysing HC mobilization, blood was sampled before, on day 3 and 5 after G-CSF injection. Blood for engraftment checks was sampled at days +20 (+30 for NSG model), day+60 and day+125 after HCT. Spleens were harvested at day+125 and meshed through a 40µM cell strainer rinsed with isolation buffer (PBS + 2% FCS + 1 mM EDTA) for cell isolation. BM cells were harvested on day+125 by flushing of tibia and femur with isolation buffer through a 23G needle and subsequent filtering through a 70 μm cell strainer. After erythrocyte lysis (150 mM NH4Cl + 10 mM KHCO3 + 0.1 mM Na2EDTA), PB, splenic and BM cells were stained for 30 minutes with the following flow cytometry antibodies in MACS buffer (PBS + 0.5% BSA +1 mM EDTA): anti mouse H2kb-PE (1:100), Gr1-PE-Cy7 (1:200), CD80-APC (1:200), CD45-FITC (1:50; all Biolegend, USA), CD11c-PE (1:100; Invitrogen, USA), B220-PerCP-Cy5.5 (1:200), H2kd-FITC (1:50), CD3-APC (1:200), CD4-PE-Cy7 (1:200), CD8-APC-Cy7 (1:200) and CD11b-APC-Cy7 (1:200, all BD Biociences, USA). In NSG trials, the following anti-human antibodies were applied: CD3-APC (1:20), CD4-PerCP-Cy5.5 (1:20), CD8-APC-Cy7 (1:40), CD45-PE-Cy7 (1:20; all Biolegend, USA). Cells were detected with a FACSCanto II with BD FACSDiva™ Software v8.0.2 and the data was analyzed with FlowJo 10.6.1 Software (TreeStar Inc., Ashland, OR, USA).

Positive engraftment was defined as a minimum of 85% murine donor cells in the CD3+ fraction of the BDF1-recipients’ blood at day+20, respectively the presence of human CD3+ cells in peripheral blood at d+30 after HCT and a minimum of 1% of human CD3+ cells or > 5% human CD45+ cells in the spleen of NSG mice upon the day of experiment abortion.

### Mouse serum analysis

Blood from BDF1 and NSG mice was sampled by retro orbital bleeding under anesthesia. Blood was left to clot at room temperature 30 minutes and centrifuged to separate the serum fraction. Serum was analyzed with the LEGENDplex™ Mouse Inflammation Kit (Biolegend, USA) according to the manufacturer’s protocol. Readings of the multiplex assays were performed at the BD FACS Aria II.

### Statistics

Survival data were analyzed using the Kaplan–Meier method and the Mantel–Cox log-rank test. For analysis of all other data, unpaired Student’s *t*-test was used, unless indicated otherwise. Error bars represent mean ± standard error of the mean (SEM). Values of *P ≤ 0.05 were considered statistically significant, with **P<0.01, ***P<0.001 and **** P<0.0001. All statistical analyzes were performed using GraphPad Prism software (GraphPad Software Inc., La Jolla, CA, USA).

## Results

### Mobilization of HCs by G-CSF treatment in mice

**Figure 1:**
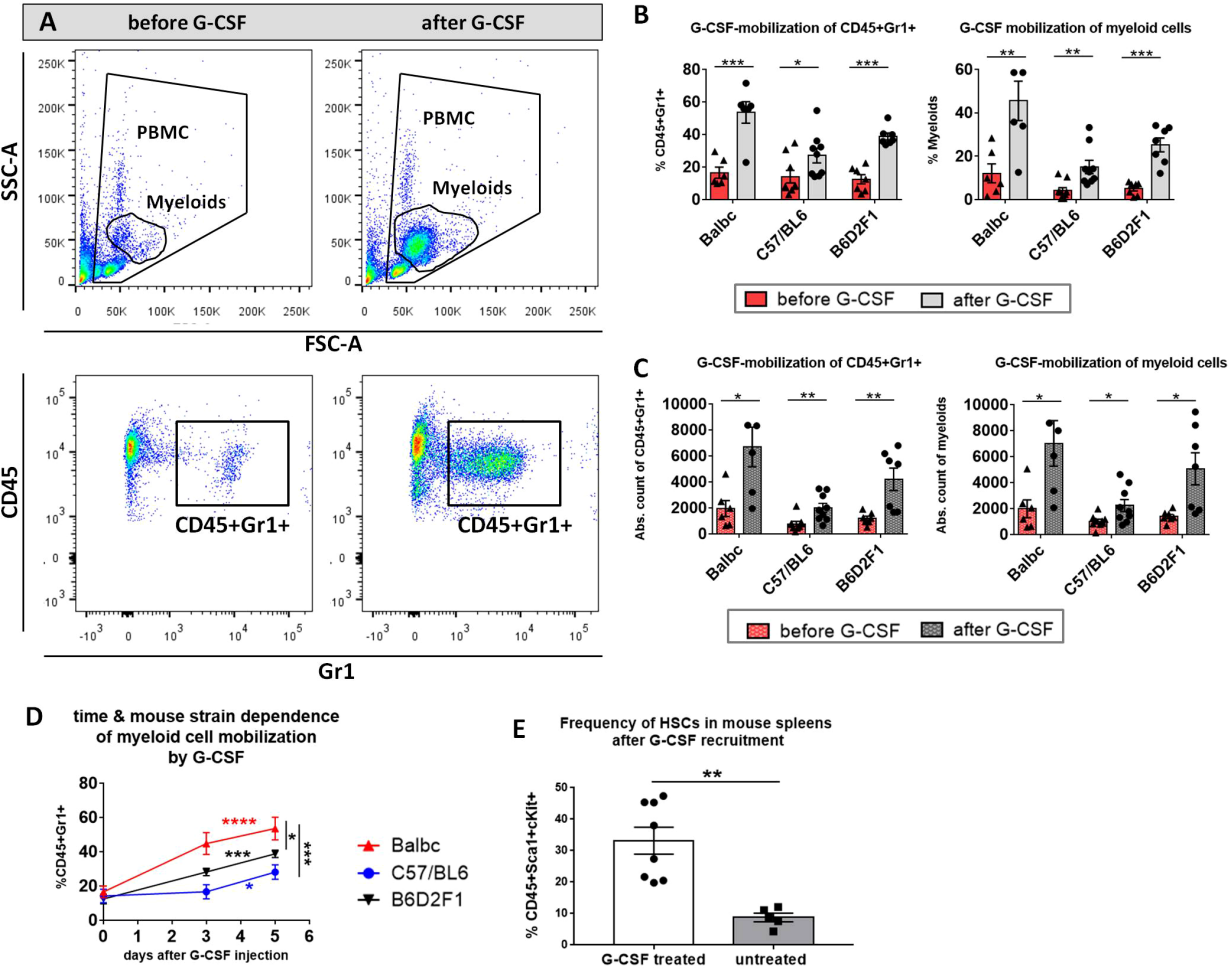
Myeloid cell-mobilization by G-CSF in peripheral blood of Balb/c, C57BL/6 and B6D2F1 mice. **(A)** FACS gating strategy of unstained myeloid cells in FSC/SSC and of stained CD45+Gr1+ cells. Example of a visible increase in myeloids and CD45+Gr1+ cells after 5 consecutive days 10 µg G-CSF injection in a BDF1-mouse. **(B)** Significant increase in % CD45+Gr1+ and % myeloids and **(C)** in the absolute cell count of CD45+Gr1+ and myeloids in the peripheral blood of different mouse strains before and after 5 day G-CSF treatment. **(D)** Temporal rise of % CD45+Gr1+ cells in the peripheral blood of different mouse strains from day 0 to 5 of G-CSF injection. Balb/c: n=6, BDF1: n=7, C57BL/6: n=9. **(E)** Frequency of CD45+Sca1+cKit+ HCs in the spleen of untreated vs. G-CSF treated mice. Cell frequencies were determined from isolated FACS-stained whole spleen PBMCs with or without 10 µg G-CSF treatment for 5 days. Pooled BDF1 (n=3), Balb/c (n=2) and C57BL/6 (n=3). Data pooled from three independent experiments. Error bars indicate mean+SEM. *P<0.05, **P<0.01, ***P<0.001, ****P<0.0001 by Holm-Sidak test **(B, C)** and unpaired students t-test with α=0,05 **(D, E)**.

To determine the yield of donor HCs mobilized by G-CSF treatment, examine possible temporal differences in the recruitment of HCs between distinct mouse strains and to optimize the mobilization protocol in our B6→ BDF1 model, blood was sampled from B6- and BDF1-donors before G-CSF injection, at day 3 and day 5 after injection and analyzed by flow cytometry for the total cell count and the frequency of CD45+Gr1+ myeloid subset in PB (**Figure 1**). Flow cytometry highlighted a visible increase of myeloid cells, which were determined by staining of CD45+Gr1+ after 5 days of injection (**Figure 1A**). Cell frequencies (**Figure 1B**) as well as absolute cell counts (**Figure 1C**) of CD45+Gr1 positive cells and unstained myeloids were significantly increased after 5 day G-CSF treatment in all three mouse strains. Nonetheless, there was a time- and strain-dependence of HC-recruitment: CD45+Gr1+ cells were recruited more efficiently in Balb/c mice compared to BDF1 and C57BL/6 mice, which showed slowest rise in cell frequency after 5 days of G-CSF treatment. While CD45+Gr1+ cell frequencies were more than doubled in Balb/c mice already at day 3 of G-CSF injection, BDF1 mice recruited cells in a linear manner, reaching maximum numbers around day 5. C56/BL6 showed only slightly elevated CD45+Gr1 cell numbers at day 3 and reached a peak not before day 5 (**Figure 1D**). Prolonged G-CSF administration was not necessary, because sufficient engraftment of 5 day -mobilized splenocytes was verified in the G-CSF-dependent models.

After 5 days of 10µg G-CSF recruitment, 33.22% CD45+Sca1+cKit+ HCs accounted of all spleen PBMCs in G-CSF treated mice, while untreated wild type mice harbored only 8,76% of the same population (**Figure 1E**).

### Cell dose dependency on cGvHD development after allo-HCT in B6D2F1-recipients

**Figure 2:**
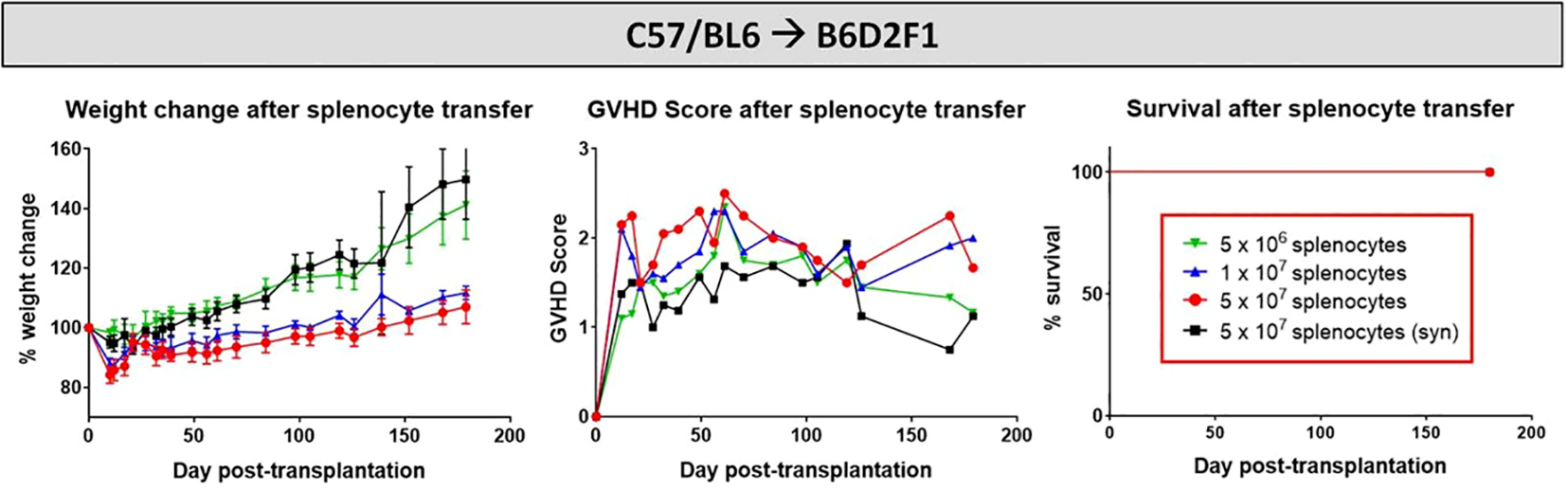
Cell dose titration for establishment of cGvHD in mouse models. C57BL/6 ➔ BDF1 model running for 180 days. Three different cell doses (1x106, 1x107 and 5x107) were transplanted in allo-setups and one as syn-control. Clinical GvHD was estimated by weight change (left), GvHD score (middle) and survival (right) Animals were regularly scored for five clinical parameters (weight loss, posture, activity, fur and skin) on a scale from 0 to 2. Clinical GvHD score was summarized of all five parameters. Error bars show mean ± SEM. n=5 per group.

Aiming to develop a stable cGvHD phenotype in the C57BL/6→BDF1 model, variable numbers of splenocytes were transplanted into irradiated recipients, which were frequently scored with onset of first GvHD signs and transplant-engraftment was determined around day +20. Criteria for a successful transplantation with GvHD signs were defined the following: (1) development of moderate weight loss (<20%), (2) a moderate average GvHD score of 2 - 4 out of 6 maximum scoring points, (3) low mortality rates and (4) detection of >85% donor cells and <15% of recipient cells in the peripheral blood of recipients around day +20 after transplantation (engraftment). In the model C57BL/6→BDF1 the highest cell dose of 5x10^7^ splenocytes fulfilled all criteria, showing a moderate GvHD score, 100% survival and a reduced weight change compared to syn-controls or lower transplanted cell doses (**Figure 2**). Flow cytometry analysis of blood measured 99.8% H2^b^-positive CD3+ cells from donors in all of the 5 recipients. In lower cell doses of 1x10^7^ (5x10^6^), only 3 out of 5 (1 out of 5) recipients showed >85% H2^b^+CD3+, while mixed chimerism was detected in two (one) mice, disqualifying them for further experiments.

### Characterization of clinical cGvHD in the C57BL/6 ➔ BDF1 model

**Figure 3:**
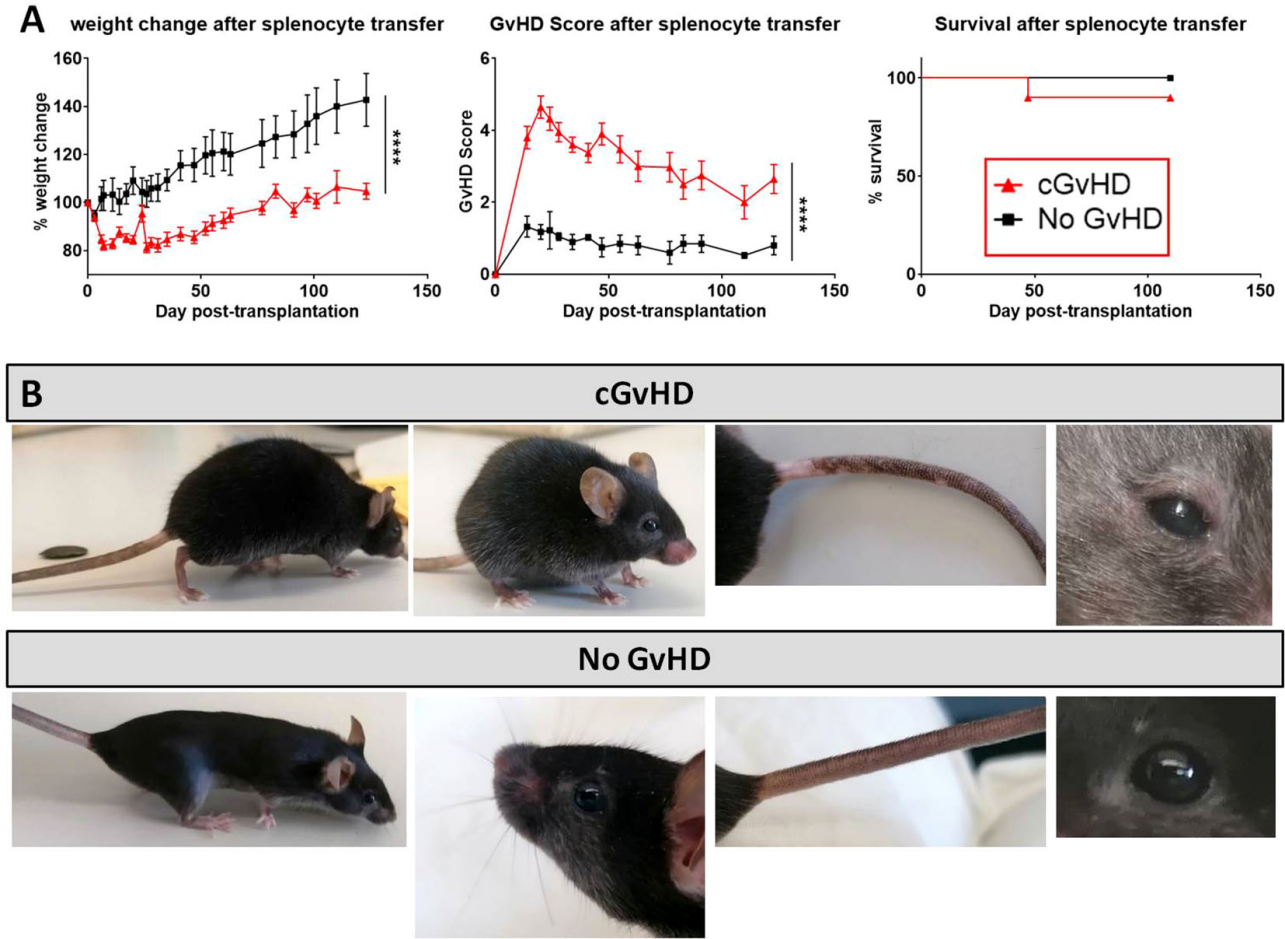
cGvHD morbidity and mortality over 125 days in C57BL/6 → BDF1. **(A)** Clinical cGvHD was estimated by frequently assessing weight change (left), GvHD score (middle) and survival (right). Animals were regularly scored for five clinical parameters (weight loss, posture, activity, fur and skin) on a scale from 0 to 2. Clinical GvHD score was summarized of all five parameters. Error bars show mean ± SEM. n=10 per group. Representative data from one out of four experiments. ****P<0.0001 by unpaired students t-test. Survival was tested by Mantel-Cox-log-rank test. **(B)** Frequently observed clinical manifestations of cGvHD in BDF1-recipients (upper): Kyphosis, ruffled fur, alopecia, skin-depigmentation and blepharitis were seen in allo-transplanted animals, while syn-mice did not show any cGvHD-symptoms (bottom).

After cell dose evaluation, recipients were transplanted with 5x10^7^ allogeneic or syngeneic splenocytes and the developing cGvHD symptoms were monitored over 125 days, before tissues were harvested and histological and genetic analysis on the disease manifestations were conducted. Allo-transplanted cGvHD mice showed a significantly reduced weight gain and greater morbidity in contrast to the syn-transplanted control group, while the overall survival was not varying between groups. GvHD developed around day +20 in an acute form with high scores (~4.5) and progressed from day +60 with moderate scores of ~3 as robustly manifested cGvHD (**Figure 3A**). As depicted in **Figure 3B**, cGvHD mice (upper row) evinced diverse clinical cGvHD symptoms around day +120, which were not detected in syn-controls (bottom row): Posture and activity: a hunched, kyphotic posture with impaired movement and a stilt walk Fur: ruffled, erected fur and loss of whiskers, alopecia. Skin: erythema with scaling, small lesions, especially of hairless areas as mouth, ears or tail. Depigmentation of tail-skin. Eyes: blepharitis and dry, opaque eye lenses.

### Clinical and histological manifestations of cGvHD in the C57BL/6➔BDF1 mouse model

**Figure 4:**
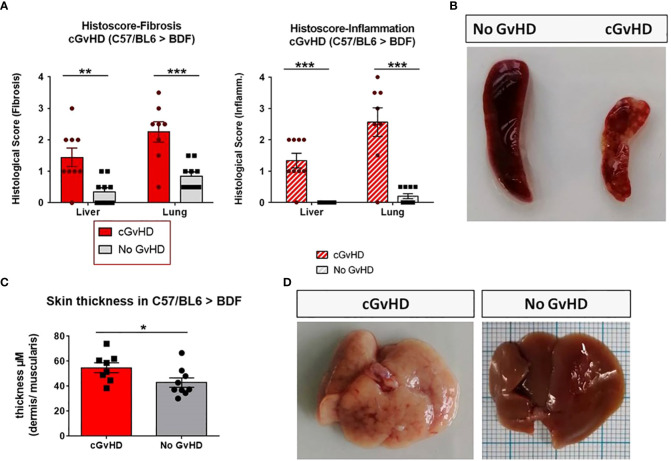
Clinical organ manifestations of cGvHD in C57BL/6➔BDF1 at day +125 after allo-HCT. **(A)** Histological quantification of fibrosis and inflammation in liver and lung of each individual mouse, based on histo-scoring ranging from 0=no to 4= severe fibrosis/inflammation. Error bars show mean ± SEM. n=9 (cGvHD); n=10 (No GvHD). Representative data from one out of four experiments. *P<0.05, **P<0.01, ***P<0.001by unpaired students t-test. **(B)** Spleen of cGvHD vs. non-GvHD mouse at d+125 after transplantation with visible sclerotic and apoptotic changes in cGvHD. **(C)** Skin thickness was increased in cGvHD vs. non-GvHD. Thickness was estimated by measurement of dermis-muscularis-ratio in Masson’s Trichrome stainings. Error bars show mean ± SEM. n=8 (cGvHD); n=9 (No GvHD). Representative data from one out of four experiments. *P<0.05 by unpaired students t-test. **(D)** Livers of cGvHD vs. non-GvHD mouse at d+125 after transplantation with visible cirrhotic changes in cGvHD.

The clinical cGvHD appearance in the C57BL/6→BDF1 model was correlated to analysis of Masson’s trichrome staining, which was utilized to quantify fibrosis, immune cell infiltration and overall epithelial damage in cGvHD target organs. Organ fibrosis as well as tissue inflammation (**Figure 4A**) of liver and lungs were quantified to be considerably severe in the allo- than in the syn-transplanted group. In the skin the thickness of the dermis related to the underlying muscularis was elevated in cGvHD animals compared to controls (**Figure 4C**). Spleens of cGvHD mice (**Figure 4B**) were marked of sclerotic tissue loss and necrotic foci and were notably smaller in size than spleens of syn-mice due to cellular apoptosis. cGvHD livers (**Figure 4D**) appeared pale, with intrahepatic bleedings and necrotic areas, emerging as cirrhotic changes, which were not detected in non-GvHD animals.

**Figure 5:**
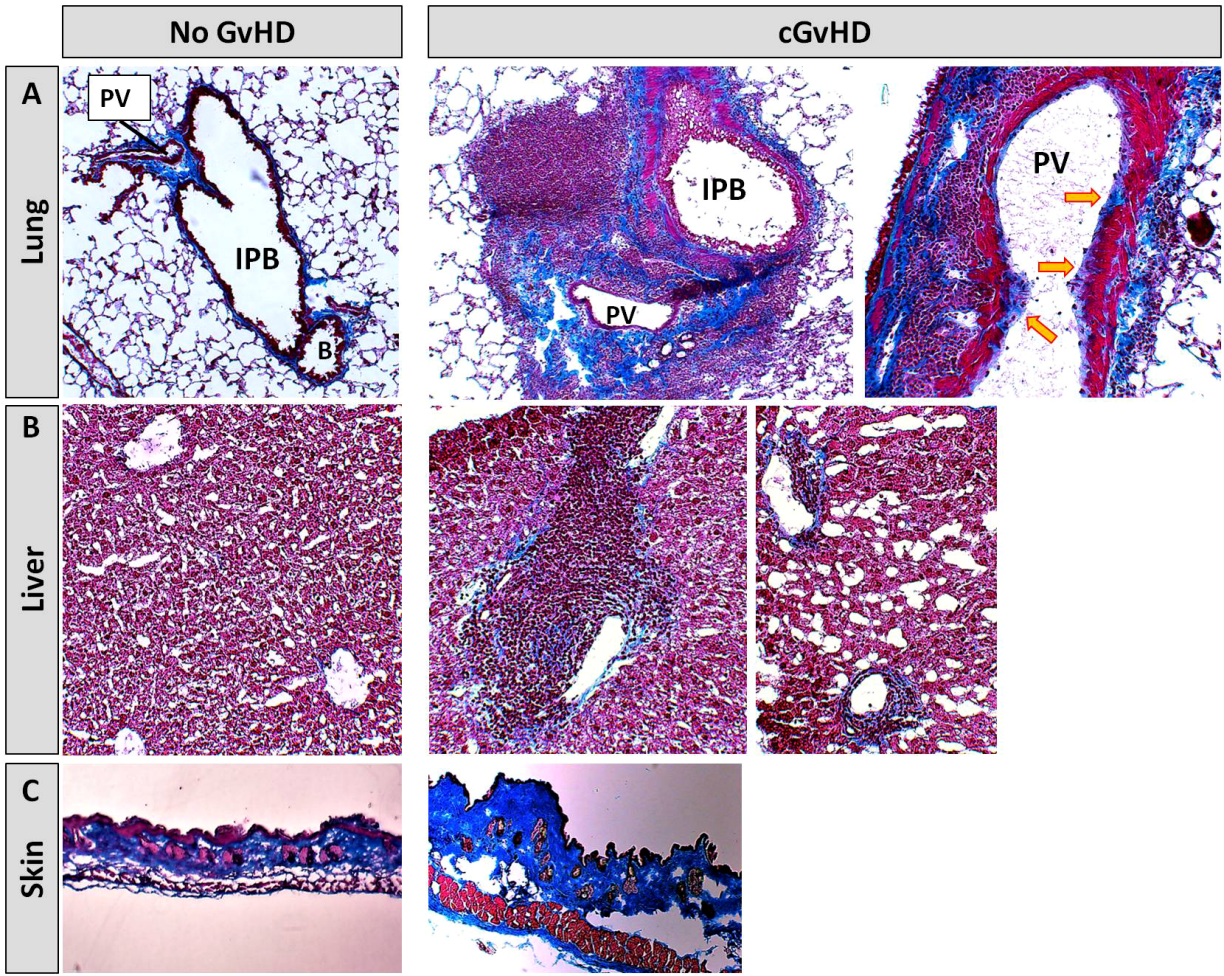
Histological characterization of murine cGvHD at day+125 after allo-HCT. Masson’s trichrome fibrosis staining in cGvHD target organs **(A)** lung, **(B)** liver and **(C)** skin of syn- and allo-transplanted BDF1-recipients. Blue, collagen fibres (fibrosis); red, cytoplasm; keratin, muscle fibers and erythrocytes, dark red/black, nuclei; infiltrating immune cells. 20x magnification. PV, Pulmonary vein; IPB, Intrapulmonary bronchus; B, Bronchiolus.

In the Masson’s Trichrome staining, cGvHD lungs (**Figure 5A**) showed a massive immune cell infiltration around intrapulmonary bronchi (IPB) and pulmonary veins (PV). Infiltrates were surrounded by heavy collagen depositions, lining especially veins and small arteries, while syn-controls showed only minor collagen fibers around PV. The endothelial lining of large PV in cGvHD appeared superimposed by a visible blue collagen layer (yellow arrows), indicating an Endothelial tomesenchymal transition process in cGvHD mice. While syn-livers (**Figure 5B**) showed normal hepatic architecture without significant collagen emplacement, cGvHD manifested in marked portal vein inflammation, interspersed with fibrotic tissue. Fibrosis and inflammatory infiltrates were also detected around bile ducts and hepatic arteries. In perihepatic areas, superficial tissue exhibited remarkable hepatic steatosis. Cutaneous cGvHD occurred in the allo-HCT group (**Figure 5C**), with thickening, perivascular inflammation and sclerosis of the dermis. Single animals also exhibited vacuolization due to collagen degradation in dermal layers or showed epidermal desquamation.

**Figure 6:**
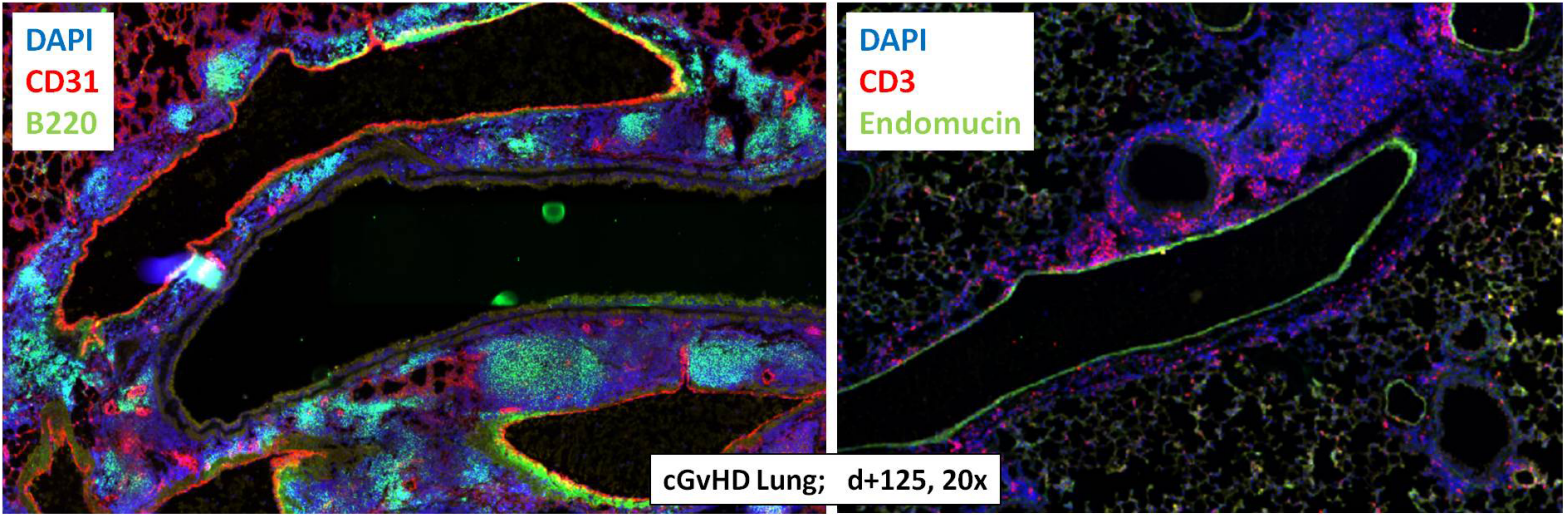
Tissue immune cell infiltration in lungs from C57BL/6 ➔ BDF1 during established cGvHD. Representative immunohistofluorescence images of cGvHD lungs at day +125 showed massive immune cell infiltrates (indicated by DAPI staining, blue) with dense foci of B220+ B cells (left, green) and CD3+ T cells (right, red). Magnification 20x.

With the objective to corroborate our findings from the Masson’s stains, to characterize the extent of tissue inflammation in cGvHD target organs and to analyze differences between cGvHD and non-GvHD groups, additional immunohistofluorescence stainings on B- and T cells were performed. **Figure 6** exemplarily describes massive immune cell accumulations in cGvHD lungs from allo-BDF1-recipients, which harbored dense B220+ B cell (left) and CD3+ T cell infiltrates (right). Similar results were also found exclusively in other examined cGvHD target organs, as liver, colon, skin and eyes but not in tissues from syn-recipients.

**Figure 7:**
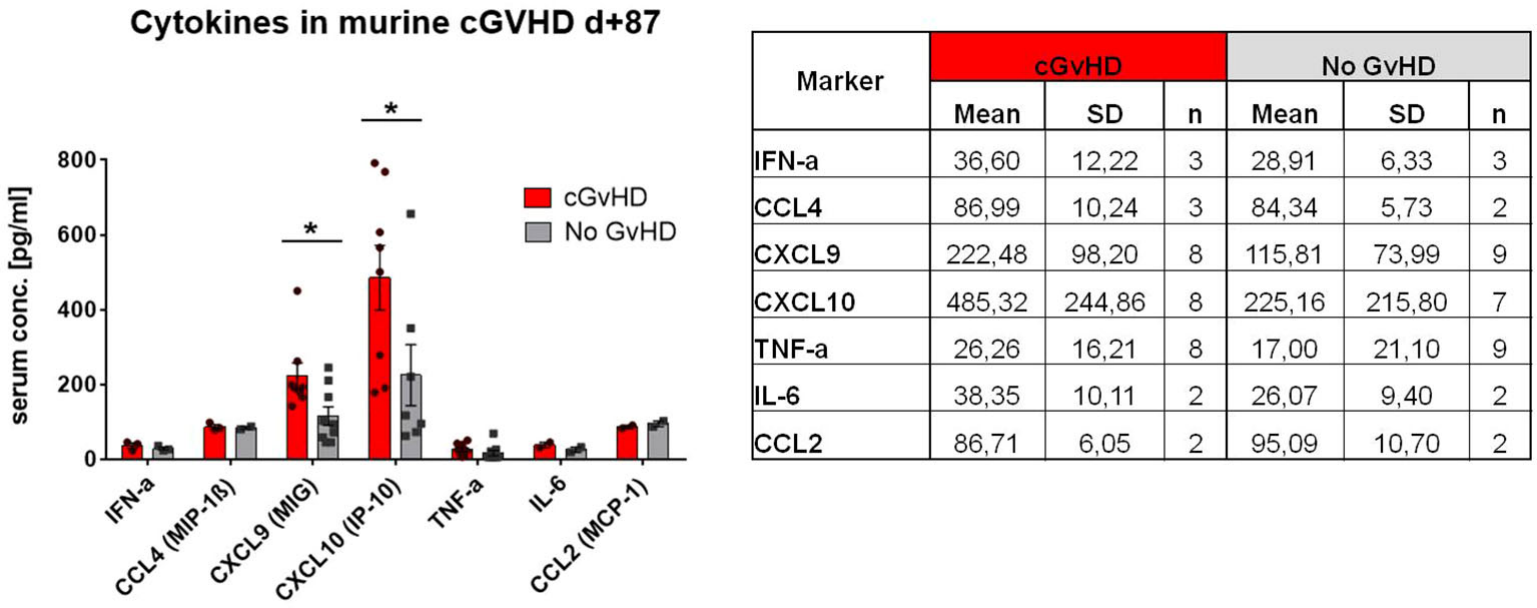
Cytokine levels in serum of cGvHD vs. non-GvHD in C57BL/6➔ BDF1 model at day+87 after transplantation. Table shows mean+SD for each analyzed marker. Graph illustrates cytokine serum concentration in pg/mL. Error bars show mean ± SEM. Representative data from one experiment. *P<0.05, by unpaired students t-test.

Due to the massive inflammatory cellular involvement in cGvHD target organs we hypothesized, that also systemic cytokine levels are affected, expecting especially higher levels of pro-inflammatory cytokines in serum during disease. We partly confirmed our assumption by detection of significantly increased levels of CXCL9 and CXCL10 exclusively in cGvHD-mice (**Figure 7**). Both markers are also frequently observed to be elevated in clinical cGvHD in patients. Other pro-inflammatory cytokines e.g. IFN-a, TNF-a or IL-6 were increased in cGvHD in tendency, but only few samples gave measurement results for these markers due to low cytokine threshold concentrations.

### Engraftment of donor cells and immune reconstitution after allo-HCT in C57BL/6 B6D2F1

**Figure 8:**
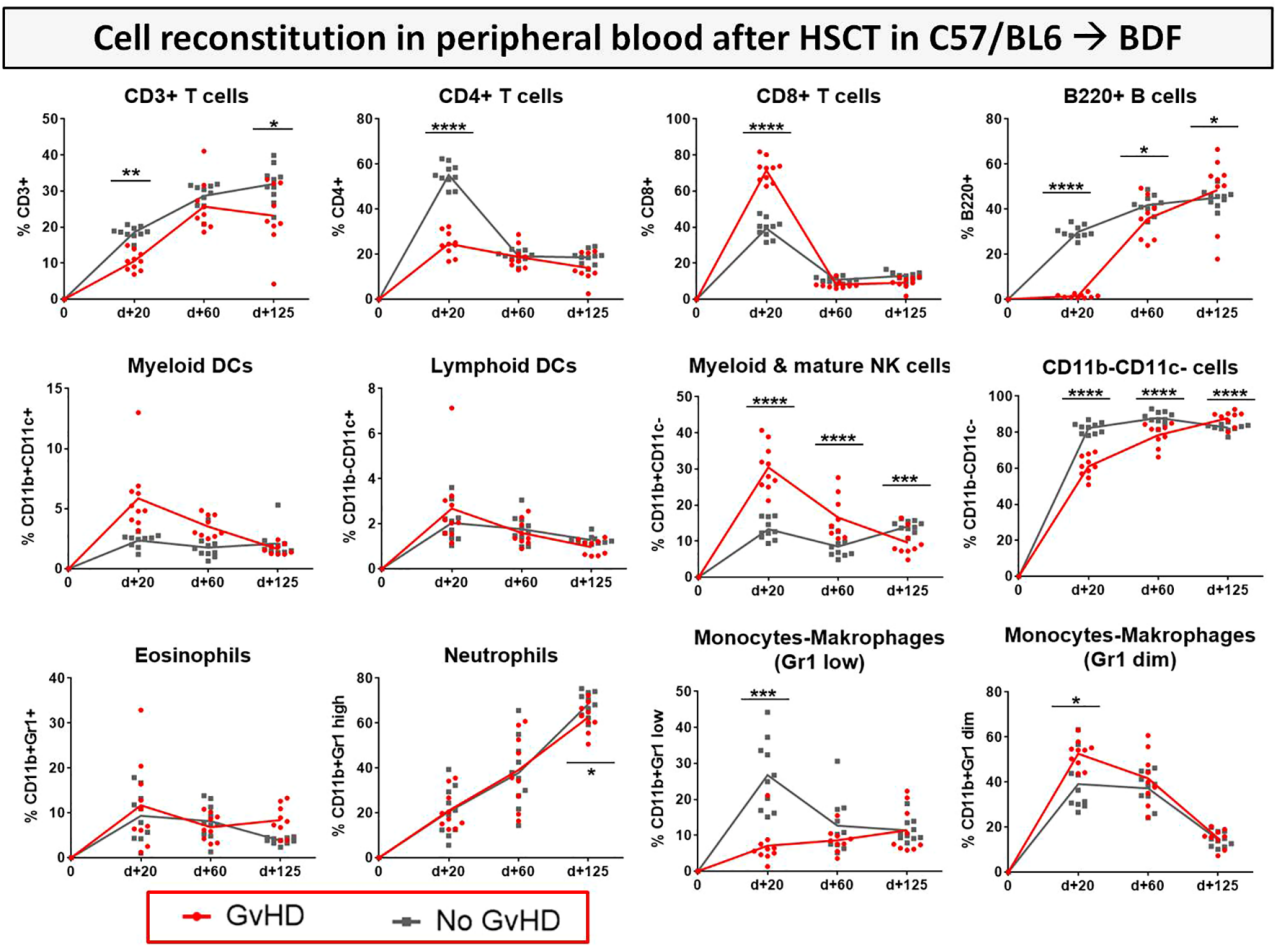
Immune cell reconstitution in peripheral blood of BDF1-recipients over 125 days after allo- vs. syn HCT. Tail vein blood was collected at three time points, PB cells were isolated, stained with the respective FACS-antibodies and samples were analyzed with flow cytometry. DCs, dendritic cells; NK, natural killer cells; dim, diminished. n=9 (cGvHD); n=10 (No GvHD). Representative data from one out of four experiments. *P<0.05, **P<0.01, ***P<0.001, ****P<0.0001 by unpaired students t-test.

After transplantation, donor syn- or allo-HCs engrafted in myeloablative irradiated recipients. To address the question how immune reconstitution is altered in the BDF mouse model and if there are differences in immune cell recovery between syn- and allo-transplanted recipients, various immune cell subsets were tracked with flow cytometry of recipients blood over 125 days (**Figure 8**). With the onset of cGvHD around d+20, reconstitution of CD3+ and CD4+ T cell subsets was significantly decelerated in cGvHD animals, while especially CD8+ CTLs were massively expanded at d+20 in this group. Reappearance of B cells in cGvHD was first detected at day+60, while B cell numbers in cGvHD mice exceeded numbers in non-GvHD controls at day+125. At d+20 and d+60, CD11b+CD11c- myeloid and mature NK cells were elevated in the cGvHD-group, but cell numbers intensively dropped until d+125, reaching lower levels than the control-group. We detected a higher proportion of Gr1+ ^diminished/high^ and vice versa less Gr1+ ^low^ monocytes and macrophages in cGvHD mice than in controls at GvHD onset but no different cell numbers between cGvHD- and control group in late established cGvHD.

**Figure 9:**
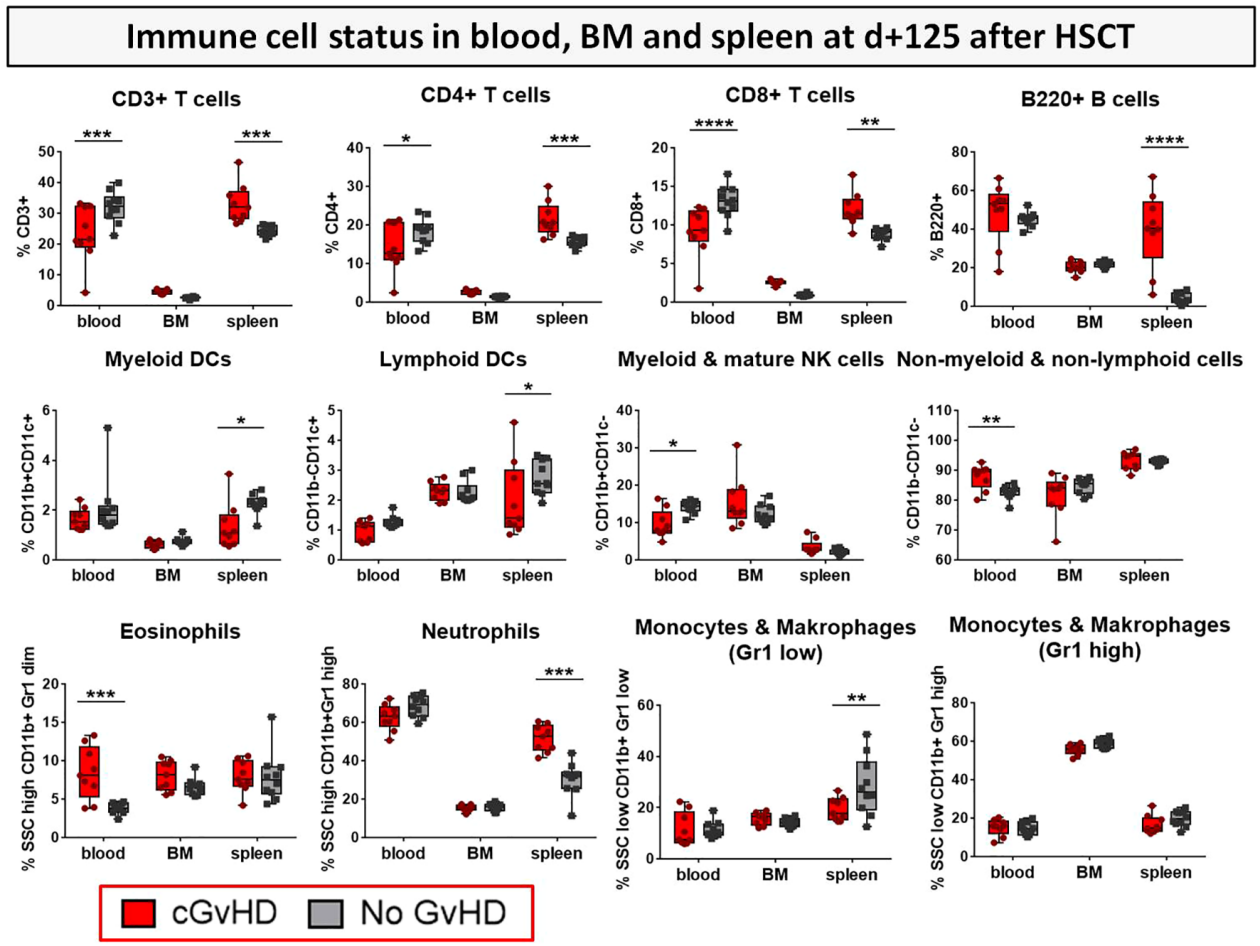
Immune cell status in peripheral blood, bone marrow and spleen of BDF1-recipients at day +125 after allo- vs. syn HCT. Blood was collected retrooribitally upon finalization. Spleens were harvested and BM flushed from tibia and femur. Immune cells were isolated, stained with the respective FACS-antibodies and samples were analyzed with flow cytometry DCs, dendritic cells; NK, natural killer cells; dim, diminished.n=9 (cGvHD); n=10 (No GvHD). Representative data from one out of four experiments. *P<0.05, **P<0.01, ***P<0.001, ****P<0.0001 by unpaired students t-test.

To estimate possible differences in cGvHD-immune cell reconstitution in various lymphatic niches, immune cell subsets were analyzed in PB, BM and spleens as the experiment was terminated at day+125 (**Figure 9**). CD3+, CD4+ and CD8+ T cell subsets were decreased in the PB, but increased in spleens of cGvHD mice. cGvHD spleens were also associated with massive B cell and neutrophil loads, which were lower in controls, while the presence of different APC subsets, such as myeloid and lymphoid DCs, monocytes and macrophages was decreased in cGvHD spleens. Interestingly, immune cell levels in the BM were generally low and no differences in cell numbers were measured between cGvHD and controls, encouraging the hypothesis that mature immune cells were hosted mainly in secondary lymphatic organs.

**Figure 10:**
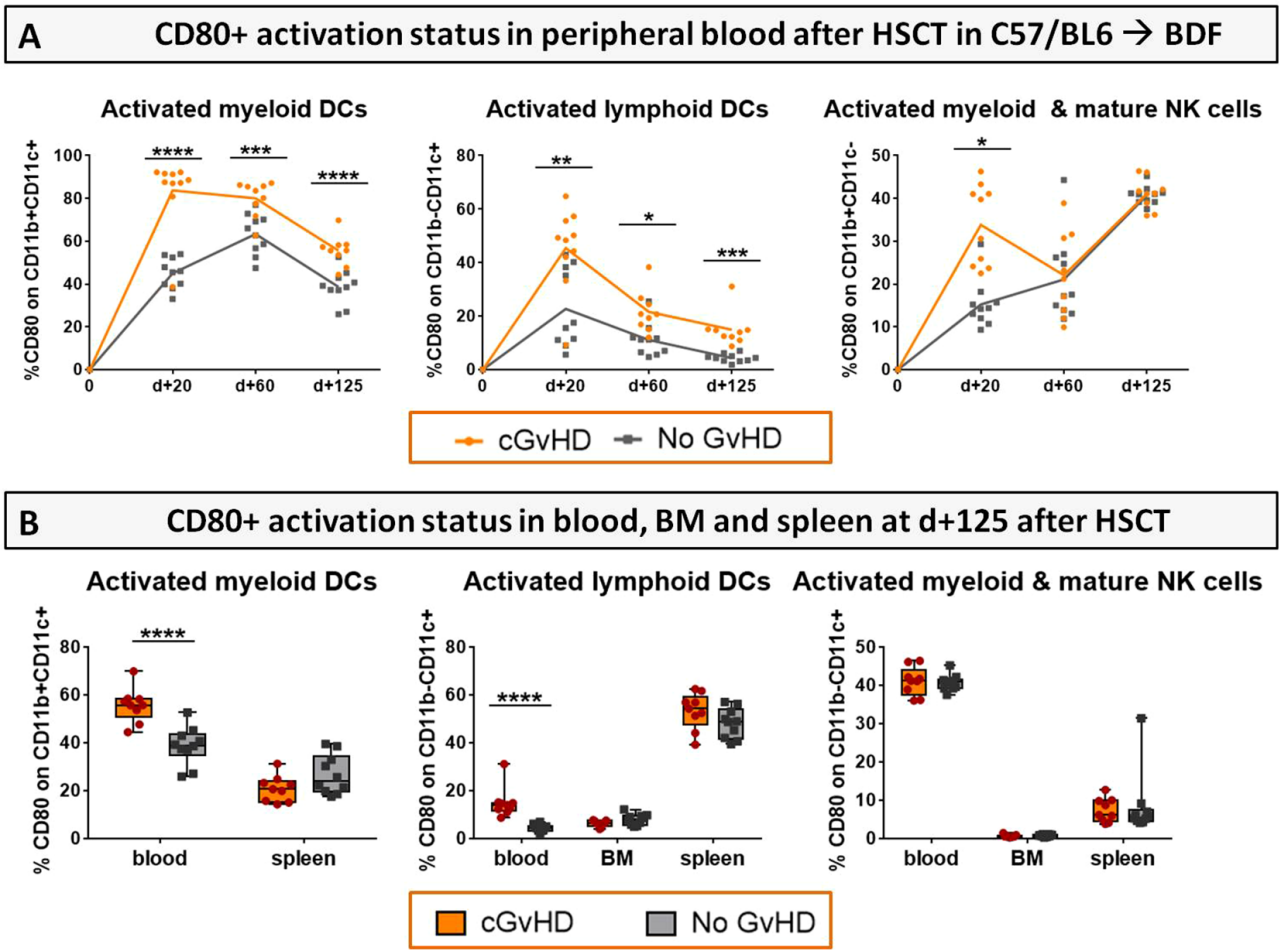
Activation status (CD80+) of DCs and NK cells in cGvHD in C57BL/6➔BDF1. **(A)** CD80+ expression on immune cell subsets in the PB over 125 days and **(B)** in PB, BM and spleen at d+125 after syn- and allo HCT. Blood was collected by tail vein bleeding or retrooribitally upon finalization. Spleens were harvested and BM flushed from tibia and femur. Immune cells were isolated, stained with the respective FACS-antibodies and samples were analyzed with flow cytometry DCs, dendritic cells; NK, natural killer cells. n=9 (cGvHD); n=10 (No GvHD).Error bars represent mean ± SEM. Representative data from one out of four experiments. *P<0.05, **P<0.01, ***P<0.001, ****P<0.0001 by unpaired students t-test.

For single populations, e.g. DCs and NK cells the progression of their activation status in the PB from d+20 to d+125 (**Figure 10A**) and in BM and spleens at day+125 after HCT (**Figure 10B**) was analyzed by estimation of the expression of co-stimulatory receptor CD80+. Mice with cGvHD harbored significantly more activated myeloid (CD11b+CD11c+) and lymphoid (CD11b-CD11c+) DCs and myeloid NK cells (CD11b+CD11c-) than control mice especially at GvHD onset. Activation levels of DCs dropped until day +125 but still stayed markedly elevated in allo-recipients. Since no differences in cellular activation in BM and spleen were revealed, we assumed the recipients circulatory, respectively the vascular system to represent the major activation site in cGvHD pathophysiology.

### Erythropoietic reconstitution during cGvHD in C57BL/6 ➔ BDF1

**Figure 11:**
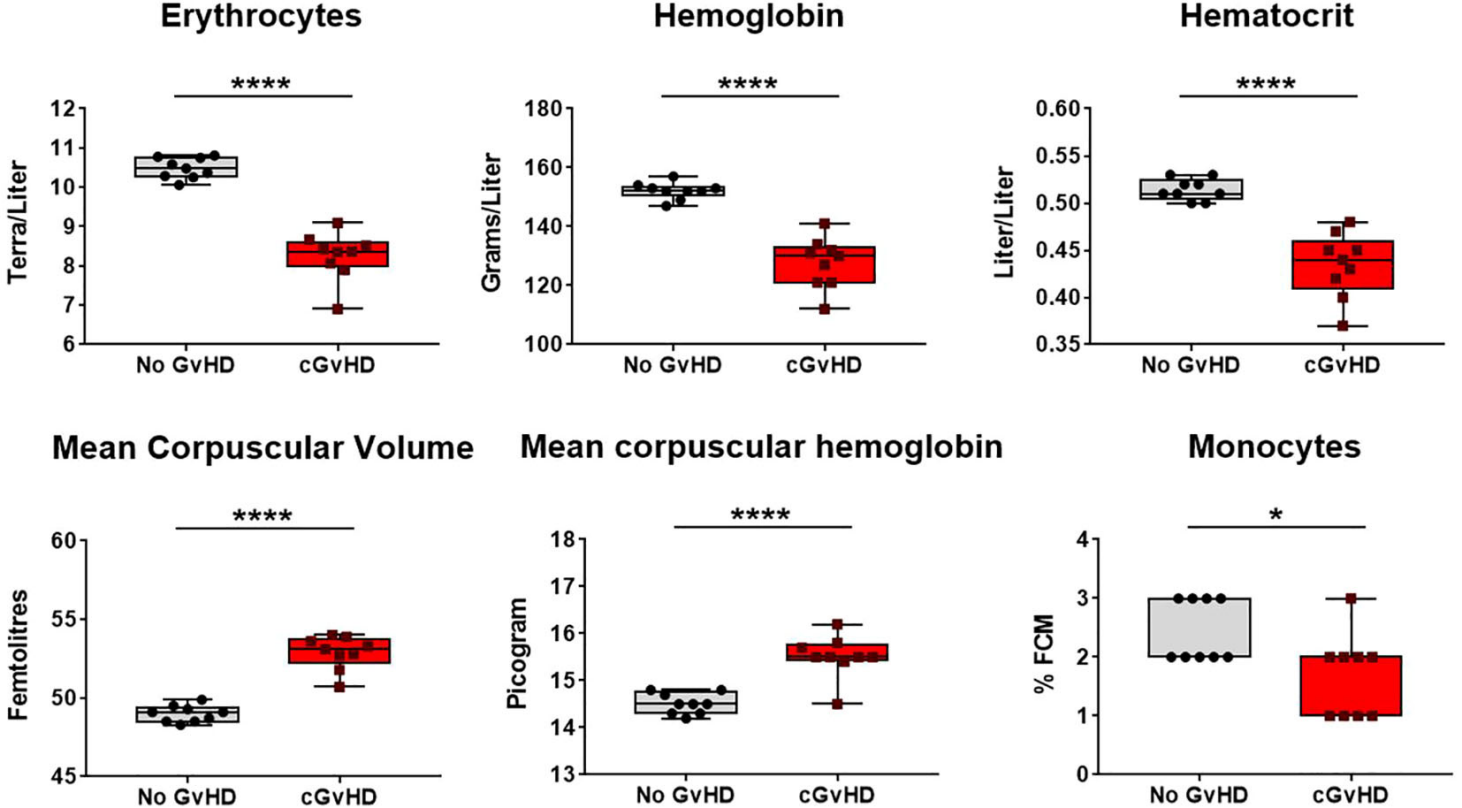
Recovery of red blood cells and related factors in cGvHD at d+90 after transplantation in C57BL/6➔BDF1. Blood was sampled from mice and differential blood count was performed by Synlab, Berlin. Data pooled from 2 independent experiments. n=9. Error bars represent mean ± SEM. *P<0.05, ****P<0.0001 by unpaired students t-test.

Aiming to investigate quantitative changes of nucleated and non-nucleated erythroid and lymphoid cells and associated features and to elucidate their relevance in cGvHD, a differential blood count was performed at day +90 in syn- and allo-HCT recipients. In cGvHD, erythrocytes, hemoglobin and hematocrit were remarkably decreased, while mean corpuscular volume and –hemoglobin were elevated, clinically presenting as macrocytic anemia (**Figure 11**; **Supplemental Figure 1**). As of nucleated cells, only monocytes were detected to be diminished in cGvHD, which corresponds to the results from the flow cytometry analyzes in PB.

### Cell dose- and huPBMC donor-dependency on cGvHD development after xeno-HCT in NSG-recipients

**Figure 12:**
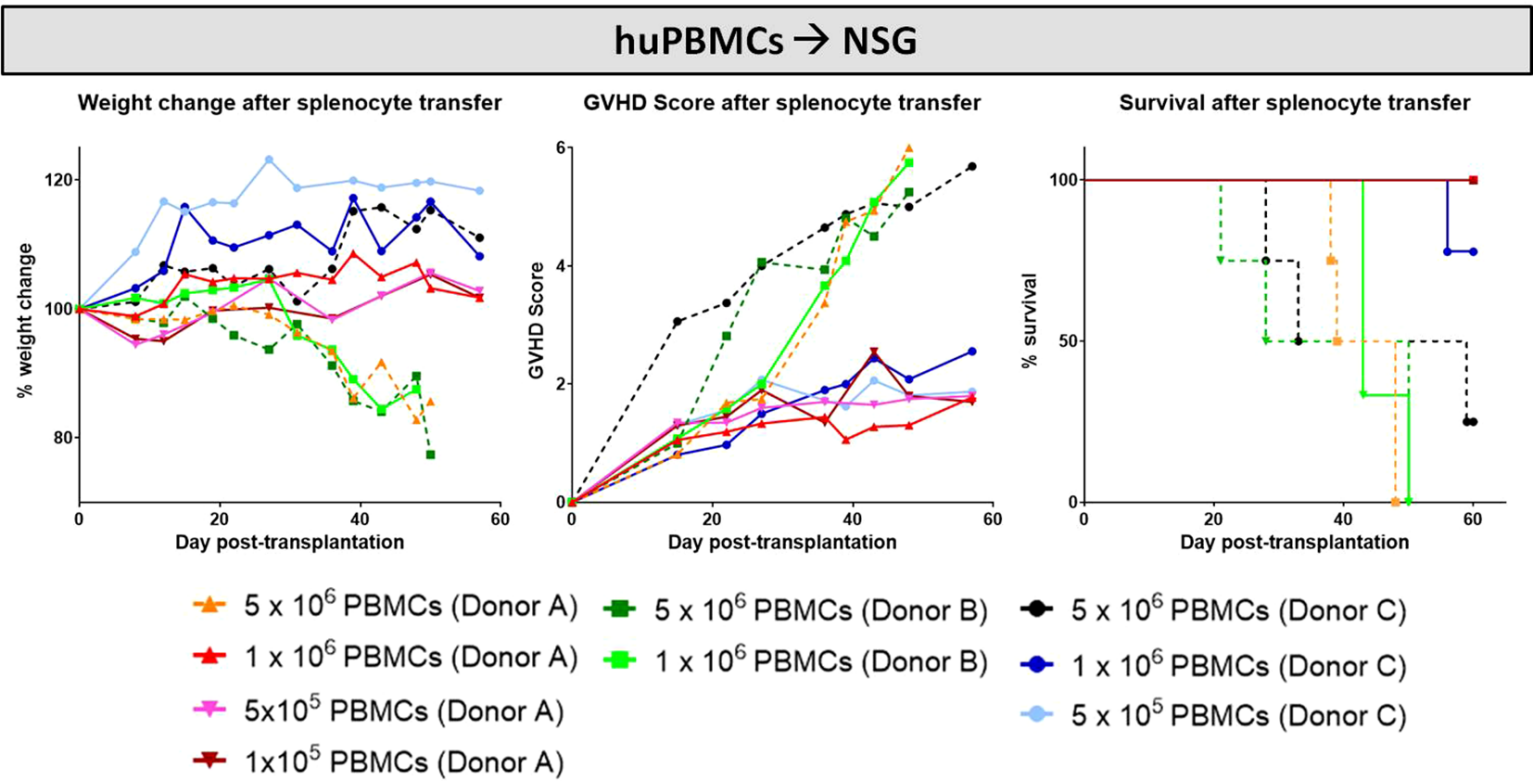
HuPBMC dose titration from three different donors for establishment of xeno-cGvHD in NSG-recipients over 60 days. Four different cell doses (5x106, 1x106, 5x105 and 1x105) from three different donors were transplanted into sub-lethally irradiated NSG. Clinical GvHD was estimated by weight change (left), GvHD score (middle) and survival (right). Animals were regularly scored for five clinical parameters (weight loss, posture, activity, fur and skin) on a scale from 0 to 2. Clinical GvHD score was summarized of all five parameters. A high variance in weight change and GvHD scores was observed not only between the cell doses, but among donors. Dotted lines indicate highest cell doses.

Similar to the murine cGvHD-models, variable numbers of huPBMCs of different donors were transplanted into sub-lethally irradiated, immunocompetent NSG mice and developing cGvHD was assessed by surveillance of weight change, GvHD score and survival. Criteria for a successful transplantation with GvHD signs were defined the following: (1) development of moderate weight loss (<20%), (2) a moderate average GvHD score of 2 - 4 out of 6 maximum scoring points, (3) low mortality rates and (4) detection of > 0.5% huPBMCs in the PB of recipients around day +30 after transplantation (engraftment) and of >5% huPBMCs in the spleen at the end of the experiment. **Figure 12** gives an overview on the donor dependency of developing cGvHD in the NSG-model: the highest transplanted cell dose (5x10^6^) resulted in high mortality and high morbidity independent from donor origin, while huPBMCs from donor C did not cause significant weight loss. Nearly no differences in weight change, GvHD scores and in survival were observed among the lower transplanted cell numbers (1x10^6^, 5x10^5^ and 1x10^5^) of donor A, indicating that it requires a minimum threshold cell number for cGvHD development. The group of 1x10^6^ transplanted huPBMCs revealed the biggest variance between donors: while mice transplanted with cells from donor A developed the mildest cGvHD with a low average score of <2, 100% survival and no weight loss, cells from donor B caused rapid weight loss, high cGvHD morbidity and 100% mortality before day 60. NSG-recipients transplanted with 1x10^6^ huPBMCs from donor C developed moderate cGvHD scores of ~3 and moderate survival rates, designating this condition as most appropriate for further experiments. To estimate, if the donor-related differences in cGvHD severity cohere with the transplanted T cell numbers, isolated donor cells were stained for CD3+ and analyzed with FACS. Of whole PBMCs, donor A comprised a high frequency of 52.8% CD3+ T cells, while donor B showed 42.0% and donor C 31.3% CD3+ T cells. Thus, the transplanted donor T cell number alone did not seem to have a direct influence on the cGvHD severity.

To evaluate if the transplanted cell amount has an influence on the engraftment, the proportion of human T cells in the PB of NSGs transplanted with three different huPBMC doses after day +30 was checked by flow cytometry. Analyzes revealed that huCD3+ T cell persistence in the PB is independent from the transplanted huPBMC dose: 12 of 13 NSG-recipients (92,3%) from the group with the highest infused huPBMC number of 5x10^6^ cells per animal were found to have successfully engrafted with an average huCD3+ T cell frequency of 29.15 ± 8.81 in the PB, while in the group of 1x10^6^-transferred cells, only 20 out of 27 recipients (74.0%) harboured a minimum of >0.5 huCD3+ T cells, with an average frequency of 14,2 ± 2,48% huCD3+ in successfully engrafted recipients. The lower transplanted huPBMC dose of 5x10^5^ resulted in full engraftment of 8 out of 9 (88.9%) and a mean percentage of 22.05 ± 0.97% huCD3+ cells in the PB of NSG-recipients.

### Characterization of clinical cGvHD in the huPBMCs➔ NSG model

**Figure 13:**
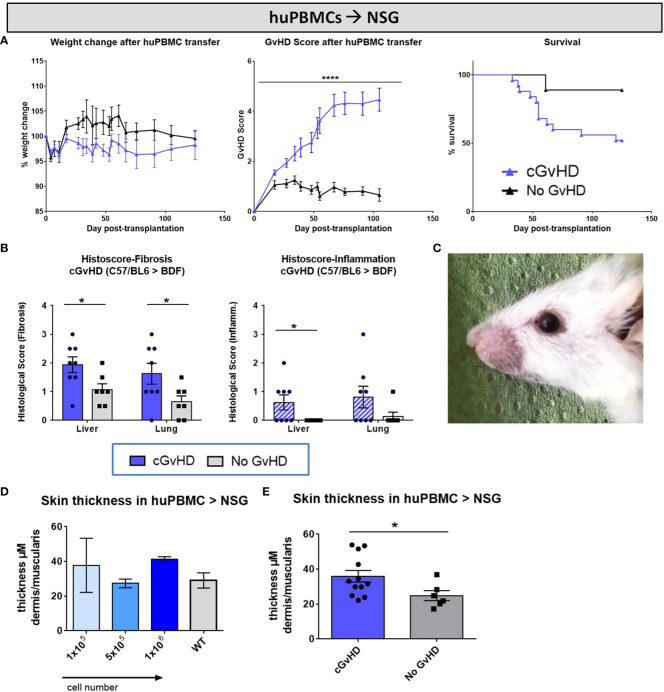
Clinical organ manifestations of cGvHD in huPBMCs➔ NSG at day +125 after allo-HCT. **(A)** Clinical cGvHD was estimated by frequently assessing weight change (left), GvHD score (middle) and survival (right). Animals were regularly scored for five clinical parameters (weight loss, posture, activity, fur and skin) on a scale from 0 to 2. Clinical GvHD score was summarized of all five parameters. Error bars show mean ± SEM. n=20 (cGvHD), n=8 (no GvHD). Representative data from one out of two experiments. ****P<0.0001 by unpaired students t-test. Survival was tested by Mantel-Cox-log-rank test. **(B)** Histological quantification of fibrosis and inflammation in liver and lung, based on histo-scoring ranging from 0= no to 4= severe fibrosis/inflammation. Error bars show mean ± SEM. n=8 (cGvHD); n=7 (No GvHD). Representative data from one out of two experiments. *P<0.05 by unpaired students t-test. **(C)** Exemplary photo of eye-involvement in cGvHD pathology. Eyes showed signs of blepharitis and dryness and opacification of lenses. **(D)** Skin thickness was not affected by varying transplanted huPBMC-doses, but was **(E)** increased in cGvHD vs. non-GvHD. Thickness was estimated by measurement of dermis-muscularis-ratio in Masson’s Trichrome stainings. Error bars show mean ± SEM. n=12 (cGvHD); n=6 (No GvHD). Pooled data from three independent experiments. *P<0.05 by unpaired students t-test.

Aiming to evaluate and describe the clinical manifestations in developing cGvHD after xeno-transplantation, one group of NSG-recipients received huPBMC-transfers of 1x10^6^ cells from donor C after non-lethal irradiation, while the ‘No GvHD’ control group was only irradiated without further cell infusion. Both groups were monitored and scored over 125 days equally to the previously described BDF1-model. While weight loss was no frequently recorded symptom occurring in xeno-cGvHD, transplanted mice exhibit steadily increasing cGvHD with scores to a maximum of ~4.5 and a higher transplant-related mortality compared to controls, emerging from day +50 and stabilizing around day+70 (**Figure 13A**). Transplanted NSG-recipients were recurrently encountered with classic cGvHD symptoms as kyphosis, reduced activity, sporadic alopecia, severe skin scaling and dryness as well as opacity of the eye lenses and blepharitis (**Figure 13C**). In histological analyzes of liver and lungs of xeno-cGvHD, significantly augmented fibrosis was detected in the majority of transplanted NSGs, while a severe inflammation was not regularly detected in all recipients, respectively was not found in xeno-cGvHD but also in few controls. The current results indicated that lungs immune cell infiltration might at least be partly induced by irradiation and further aggravated by cGvHD (**Figure 13B**). The observation of higher transplanted cell doses to not causally worsen cGvHD-morbidity until a certain cell number threshold is reached, was supported by the finding that there were no differences measured in epidermal thickness in NSGs receiving varying huPBMC numbers (**Figure 13D**), but epidermis is distinctly thickened in animals receiving 1x10^6^ huPBMCs, compared to controls (**Figure 13E**).

**Figure 14:**
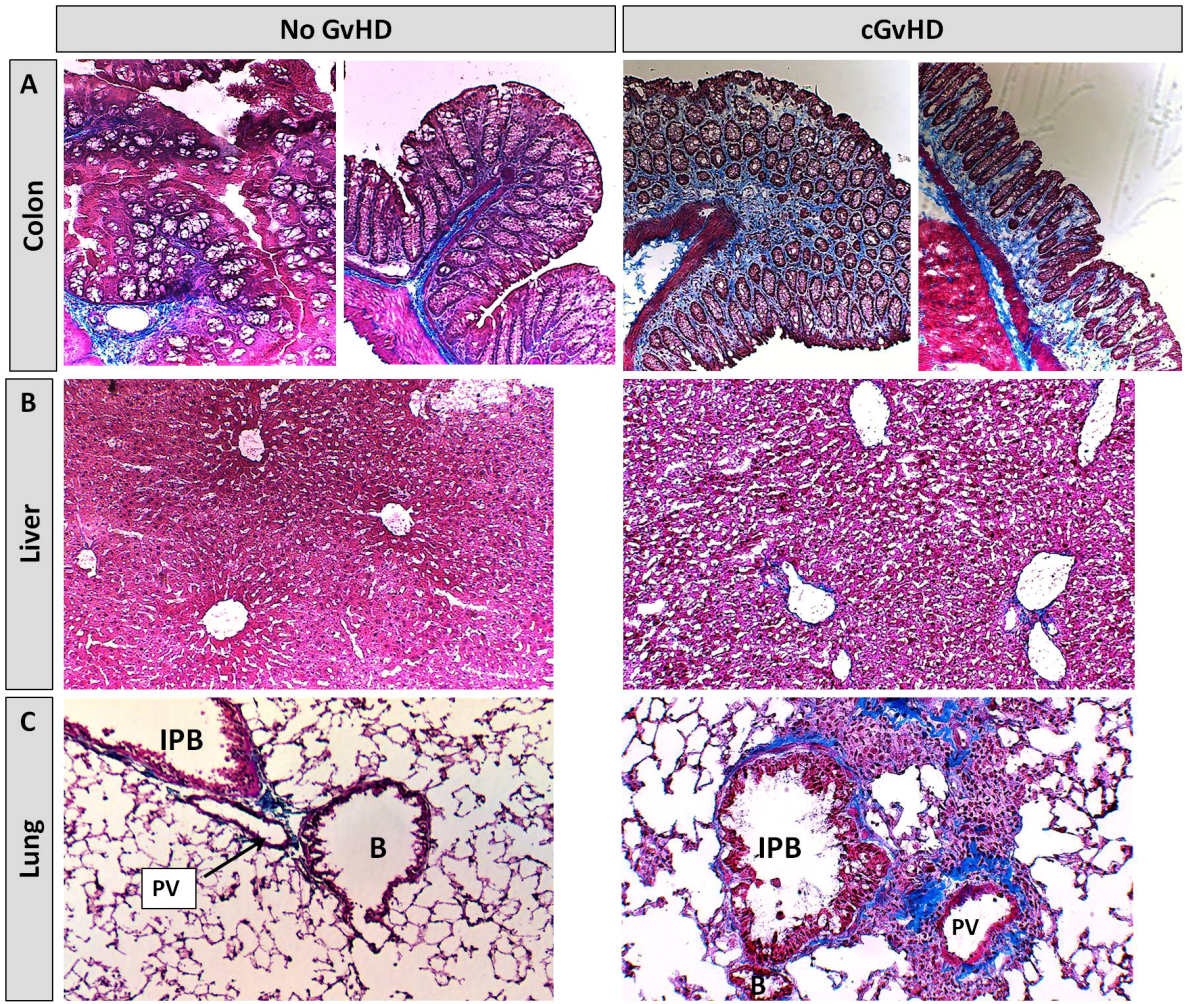
Histological characterization of xeno-cGvHD at day+125 after xeno-HCT. Masson’s trichrome fibrosis staining in cGvHD target organs **(A)** colon, **(B)** liver and **(C)** lung of xeno-transplanted and only irradiated, but not-transplanted NSG-recipients. Blue, collagen fibres (fibrosis); red, cytoplasm; keratin, muscle fibers and erythrocytes, dark red/black, nuclei; infiltrating immune cells. 20x magnification. PV, Pulmonary vein; IPB, Intrapulmonary bronchus; B, Bronchiolus.

Further cGvHD organ manifestations were illustrated performing Masson’s Trichrome fibrosis stainings. Clinical features of gastrointestinal involvement, similar to the human cGvHD pathology, were detected exclusively in cGvHD but not in controls, such as mucosa inflammation with mild crypt and gland apoptosis (**Figure 14A**). Hepatic cGvHD mainly appeared as moderate fibrotic fibre incorporations around vessels, veins and bile ducts (**Figure 14B**), with minor signs of immune cell infiltrates surrounding only bigger vascular tissues. Lungs of control-group-NSGs showed mild fibrosis around pulmonary veins and arteries without significant inflammation signs. Xeno-transplanted animals depicted altered lung parenchyma, damaged by dense infiltration-foci interspersed by fibrotic fiber-networks mainly around pulmonary veins and bigger bronchioles (**Figure 14C**).

### Engraftment of donor cells and immune reconstitution after xeno-HCT in the huPBMCs ➔ NSG model

**Figure 15:**
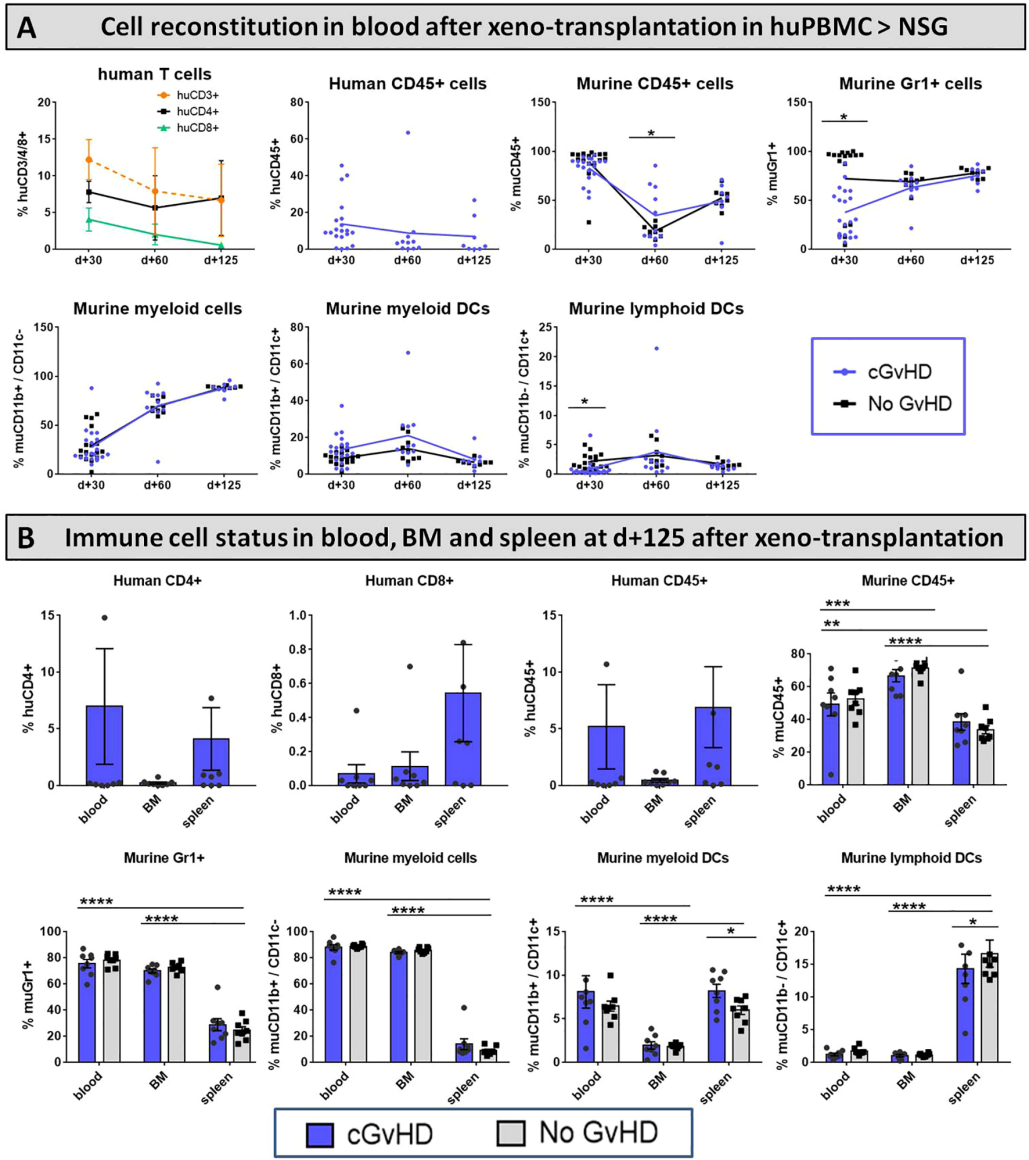
Immune cell reconstitution in **(A)** peripheral blood of NSG-recipients over 125 days and **(B)** in PB, BM and spleen at day+125 after xeno-HCT. **(A)** Tail vein blood was collected at three time points, PB cells were isolated, stained with the respective FACS-antibodies and samples were analyzed with flow cytometry. DCs= dendritic cells. **(B)** Blood was collected retrooribitally upon finalization. Spleens were harvested and BM flushed from tibia and femur. Immune cells were isolated, stained with the respective FACS-antibodies and samples were analyzed with flow cytometry DCs= dendritic cells. n=8 per group. Error bars show mean ± SEM. Representative data from one out of two experiments. *P<0.05, **P<0.01, ***P<0.001, ****P<0.0001 by unpaired students *t*-test.

Intending to track the recovery of murine immune cells after irradiation and upon cGvHD development as well as to monitor engraftment and traceability of the transferred huPBMCs in the PB of recipients, blood was sampled from NSGs periodically over 125 days after HCT and immune cell frequencies were analyzed using flow cytometry. As expected, human cell proportions, especially of CD45+ leukocytes and CD3+ and CD8+ T cell subsets, steadily decreased over the examined time, while the frequency of CD4+ T cells remained stable and from day +60 (with cGvHD onset) even slightly expanded (**Figure 15A**, upper row), indicating a huCD4+-dependent cGvHD-pathophysiology. Around day +60, also the frequency of murine CD45+ leukocytes is elevated in cGvHD, introducing the hypothesis that human T cells can be stimulated by murine tissue and vice versa activate murine immune cells or damage murine tissue in a cross-reactive manner. A remarkably decelerated reconstitution of murine Gr1+ myeloid-derived cells and CD11b-/CD11c+ lymphoid DCs was measured around day +30, but normalized to control-group levels at later stages (**Figure 15A**, bottom row).

To discern the issue, in which lymphatic organs the transplanted huPBMCs and murine donor-cells home and if there are differences in cell-locations emerging during cGvHD, various cell frequencies were analyzed in PB, BM and spleen at day+125. While huCD4+ T cells and huCD45+ leukocytes were mostly detected to circulate in the periphery and the spleen, but not the BM, huCD8+ T cells were predominantly found to reside in spleens and in small numbers in BM and PB. The majority of murine CD45+ leukocytes were found to home in the BM, indicating complete HC-reconstitution and cell-recurrence after irradiation at day+125 (**Figure 15B**, upper row). Murine CD11b+ and Gr1+ myeloid-derived cells were measured with similar higher numbers to prevail in the BM and in the PB circulatory system, while both subsets appeared in spleens to a limited extent (**Figure 15B**; bottom row).

Considerable differences of cellular reconstitution among cGvHD and control group were revealed in murine DCs of distinct origin: while the frequency of myeloid DCs was higher, lymphoid DCs were diminished in spleens of cGvHD-mice (**Figure 15B**, bottom row).

### Erythropoietic reconstitution during cGvHD in the huPBMCs NSG model

Monitoring the erythropoietic recovery after xeno-HCT, the differential blood count performed at d+90 displayed a significantly increased frequency of murine lymphocytes in cGvHD, indicating the transferred huPBMCs to effect a ‘tissue-crossing’ lymphocyte reaction in mice. Murine leukocytes, thrombocytes and neutrophils were decreased in tendency in cGvHD compared to controls, while other erythropoiesis-related factors were not affected (**Supplemental Figure 2**).

## Discussion

With the overall objective to successfully prevent and treat cGvHD after allo-HCT, the establishment and application of valuable pre-clinical mouse models is key for understanding the systemic diseases’ evolution and for translating experimental findings to the human scenario. Over the years, an extensive variety of cGvHD mouse models have been evolved, which fostered the progress of alleviating cGvHD after allo-HCT (15). However, most murine models have both, advances and limitations, so it is important to carefully characterize every single model and to evaluate, which is most appropriate to answer a particular scientific question. Examples of existing limitations are the mere sclerotic phenotype (without other typical features of human cGVHD) in some models, the short duration of 30 to 60 days and the specific target organ tropism (e.g. lung or skin) in other models. Given these limitations, our aim was to develop and characterize models which more closely resemble human cGVHD regarding the type of systemic inflammation and regarding the timing of development of the pathologies.

We established and described two relevant pre-clinical *in vivo* cGvHD models, which display a broad spectrum of the diverse cGvHD symptoms, that may be of advantage for cGvHD research and allow transferability of experimental results from bench to bedside. In the following paragraph, we discuss weaknesses and benefits of the here described haploidentical B6→ BDF1 and the xeno-transplant huPBMC → NSG models.

Most common, patients receive a stem cell infusion from a fully MHC-matched donor (35, 36), partially MHC-matched unrelated donor (37) or haploidentical donor (first-degree relatives, haploidentical in at least one set of genes) (38, 39). The latter scenario of a haploidentical HCT is used increasingly in the last years and is simulated in our B6 [H2^b^]→; BDF1 [H2^b/d^] model that represents a parent-into F1-generation, thus haploidentical immunologic disparity. The haploidentical setting may therefore be advantageous and more clinically relevant as compared with previous fully MHC-mismatched models (15, 40).

One major difference and potential improvement to other mouse models, which use the transfer of purified BM cells supplemented with purified T cells, is the mobilization of B6- and BDF1 donor-HCs with G-CSF, which allowed us to perform HCT of whole splenocytes. HC recruitment from the BM into the PB applying G-CSF is also exerted in clinical allo-HCT.

In patients, the onset of cGvHD is fluent, beginning approximately 3 months up to 2 years or even later after transplantation (41). Additionally, acute GvHD progresses to cGvHD in 70-80% of patients (42, 43). Our B6→BDF1 mouse model showed acute GvHD around d+20 after allo-HCT with spontaneous improvement afterwards. Later on, the animals developed typical cGVHD symptoms, which progressed until day+125, indicating a transformation of acute to cGVHD, as seen in many humans. Scores in the NSG model continuously increased until day+125 without an initial acute GvHD phase, but mortality rates stayed comparatively moderate. It is still not generally known, after which time cGvHD develops in mice, but the majority of published murine cGvHD models run less than 60 days, which might be too short-timed for a complete progression to robustly manifested cGvHD. The diagnosis of murine cGvHD is mainly defined by a clinical phenotype. At d+125, cGVHD mice of both of our models showed phenotypic as well as histological signs of cGvHD. Diseased NSG- and BDF1 recipients exhibited hunched posture, reduced activity, alopecia, skin scaling, erythema and ocular symptoms as dry and opaque lenses or blepharitis, all of which are frequently described to occur in cGvHD patients (32, 41, 44).

cGvHD simulates characteristics of autoimmune diseases with features of impaired immune tolerance mechanisms, like the involvement of auto- and alloreactive donor-derived T and B cells, the participation of alloantigens and mechanisms of chronic inflammation with subsequent fibrosis (32). The disease mainly manifests in the target organs skin, liver, lung and the gastrointenstinal tract. In addition, a variety of other organ systems, as mouth, joints and fasciae, muscles, eyes, the hematopoetic system or mucosal tissues may be affected (41). Our allo- as well as our xeno-HCT model depicted severe fibrosis and inflammation in the lung, liver, colon and skin of cGvHD mice. In the Masson’s Trichrome Stainings, immune cell infiltrates and fibrotic depositions as well as sclerosis were particularly found in cGvHD B6→ BDF1 recipients, where they were related to extensive tissue damage in the respective organ (e.g. epidermal thickening in the skin, hepatitis and steatosis in the liver and bronchiolitis obliterans in the lung and non-infectious colitis in the colon), with all mentioned symptoms contributing significantly to high morbidity and mortality rates after clinical allo-HCT (45–49). Our histopathological observations closely resembled the situation in patients, where fibrosis is mostly systemic, standing in contrast to the majority of published sclerotic mouse models, which mainly comprise a cutaneous pathology (15, 18). It is reported that activated monocytes, macrophages as well as eosinophils may mediate the induction of collagen production leading to systemic fibrosis (50–52). Besides, pro-inflammatory neutrophils contribute to the process of chronic pulmonary fibrosis (53), a manifestation also seen in our cGvHD BDF1 mice. In existing sclerodermatous cGvHD models, mast cell and eosinophilic cell populations have been found increased in the skin (54) and liver (55) of transplanted mice. This theory is in line with our experimental findings in cGvHD from the BDF1 model, where CD11b+Gr1+ myeloid cells and macrophages were measured to be significantly elevated in the PB until day 60 after allo-HCT and also showed signs of increased activation. Furthermore, eosinophils in the PB and neutrophils in the spleen were shown to depict elevated levels in cGvHD. Since application of G-CSF in donors is known to mobilize especially CD11b+ myelomonocytic subsets, this could be an explanation for the high frequency of aforementioned monocytic and myeloid subsets and the augmented rates of fibrosis in our cGvHD mice. Effector T cells as inflammatory triggers and macrophages and monocytes as fibrotic mediators might be recruited by the presence of chemokines, such as CXCL9 and CXCL10, which were both validated as prognostic cGvHD biomarkers in humans (56, 57) and were shown to be significantly increased in cGvHD but not in controls of our BDF1 mouse model.

In flow cytometry experiments of cGvHD BDF1 recipients, we saw a decelerated B cell recovery early as d+20 after allo-HCT during the acute GVHD phase, which is also observed in transplanted patients (58, 59). During cGVHD at d+125 after HCT, we detected massive T and B cell infiltrates in lungs and livers of cGvHD mice in flow cytometry as well as in histological immunofluorescence analyzes. It is known, that impaired B cell tolerance results in persistent activation and exaggerated proliferation of autoreactive and alloreactive B cells and contribute to the human cGvHD pathogenesis, both increasing auto-and alloantibody reactions in cGvHD patients (60, 61). Still, it has to be further elucidated, if the donor B cells in our BDF1 model are allo- or autoreactive, respectively reacting to recipient antigens exclusively or to antigens shared by donor and recipient.

Furthermore, we found an increased frequencies of CD3+, CD4+ and CD8+ T cells, B220+ B cells and neutrophils in the spleens of cGvHD mice, indicating that the observed splenic damage is evoked by inflammatory lymphocytes. While the induction of GvHD in the BDF1 mouse model was assumed to be mainly CD8+ driven, both CD4+ and CD8+ T cell subsets were not found to be significantly elevated in PB of cGvHD mice compared to controls at day+125, although clinical signs of cGvHD were detected in tissues. In a murine cGvHD chemotherapy/TBI-conditioned model Blazar et al. elegantly demonstrated that cGvHD pathology manifests in fibrosis and bronchiolitis obliterans and was associated with CD4+ as well as B220+ B cell infiltration and that germinal center B cell reactions occurring at onset of cGvHD seem to facilitate disease development. Furthermore increased frequencies of follicular T helper cells were assumed to maintain and foster germinal center B cell reactions upon cGvHD progression (62, 63). On the contrary, Zeng et al. showed cGvHD models, which described germinal center reactions as dispensible for cGvHD development, thus recently proposing extrafollicular CD4+ T- and B cell interactions to be responsible for cGvHD induction (64, 65). Given the impressive B cell aggregates that we observed in the lungs, it would be of considerable interest to determine how interactions between T cells and B cells in the spleen and target organs such as the lung contribute to the pathogenesis of cGvHD in our model.

In addition, we detected a diminished DC frequency in spleens of cGvHD BDF1 mice at day+125 after allo-HCT. This resembles the clinical situation, since severe cGvHD is associated with impaired DC recovery and reduced circulating DC numbers, contributing to failing DC-mediated tolerance in allo-HCT patients (66, 67).

Especially xenogeneic models were criticized to be highly artificial, because T cell reactivity by MHC molecules is restricted between species. Human antigen presenting cells are unable to process and present mouse MHC class II antigens to human donor T cells, making this model predominantly CD4+ T cell dependent (28). Actually, our data showed primarily human CD4+ T cells to be proliferated and circulated in the PB of NSG recipients until day+125 after HCT. Interestingly, also the proportion of murine CD45+ leukocytes at day+60 and the population of CD11b+CD11c+ myeloid DCs at day+125 was found increased in cGvHD, supporting the theory that there might be some crosstalk between murine cells, respectively tissue and human cells. Several studies indicate, that human cells can indeed recognize murine xeno-antigens in an MHC I and MHC II dependent manner and that the human CD28 T cell receptor is able to interact with its murine counterpart B7.2, facilitating co-stimulatory signals to donor T cells (68–70).

Moreover, general weaknesses occurring frequently in murine models are differences in the animal suppliers, age of mice, individual handling techniques or a homogenous microbial gut environment in mice when housed under specified, often pathogen-free conditions (71). One risk factor for development of cGvHD is an increased age of both donor and recipient (6, 72). To adapt to this situation, we used B6-donors aged more than 20 weeks and BDF1 and NSG-recipients aged at least 10 weeks. All mice were housed in open-housing cages and handled without tail fixation by tunnel or cupping techniques that might not only reduce anxiety but can help to reduce stress-related weight changes, lethargy and other factors, which can adulterate GvHD scoring results (73).

A potential point to modify in the future is the type of conditioning. Transplanted patients are often administered with cytotoxic drugs as busulfan, cyclophosphamide or fludarabine as conditioning regime prior to allo-HCT (74, 75). To secure reliable engraftment, our BDF1 model received total body irradiation, which could be prospectively refined by applying chemotherapeutic conditioning with busulfan and cyclophosphamide, e.g. as first described in pioneering work by Sadeghi et al. (76) and already further established for acute GvHD models (77).

## Conclusion

Mouse models of cGvHD can be criticized to express major differences toward the clinical allo-HCT situation, requiring careful evaluation before translating pre-clinical results into patients. On the other hand, the tight control of standardized experimental conditions and the homogenous nature of murine models, showing less variability contributed to the current knowledge about cGvHD pathogenesis and today’s consensus on treatment options. In this study, we established and characterized two improved pre-clinical cGvHD mouse models: one allogeneic, haploidentical B6→ BDF1 and a humanized PBMC→ NSG Xenograft model. Especially our well-described BDF1 model showed various clinical cGvHD manifestations as inflammation, fibrosis and an altered immunological reconstitution and could be utilized for future research on novel promising cGvHD treatment and prophylaxis strategies.

## Data availability statement

The raw data supporting the conclusions of this article will be made available by the authors, without undue reservation.

## Ethics statement

The animal study was reviewed and approved by Landesamt für Gesundheit und Soziales Berlin.

## Author contributions

LV, KR and OP designed the study. LV, KR, MK, SM, CS, JM and CF performed cGVHD experiments. LV and BJ performed histological analyzes. LV and MK performed FACS. LV analyzed data and LV and OP wrote the manuscript. All authors contributed to the article and approved the submitted version.
