# Supplementary material for: Novel pre-clinical mouse models for chronic Graft-versus-Host Disease
Source: Front Immunol. 2023 Jan 24;13:1079921. doi: 10.3389/fimmu.2022.1079921 (PMC9902926; doi:10.3389/fimmu.2022.1079921)
Supplement: Supplementary file 1 [file DataSheet_1.docx]

Supplementary Material

# Supplementary Figures


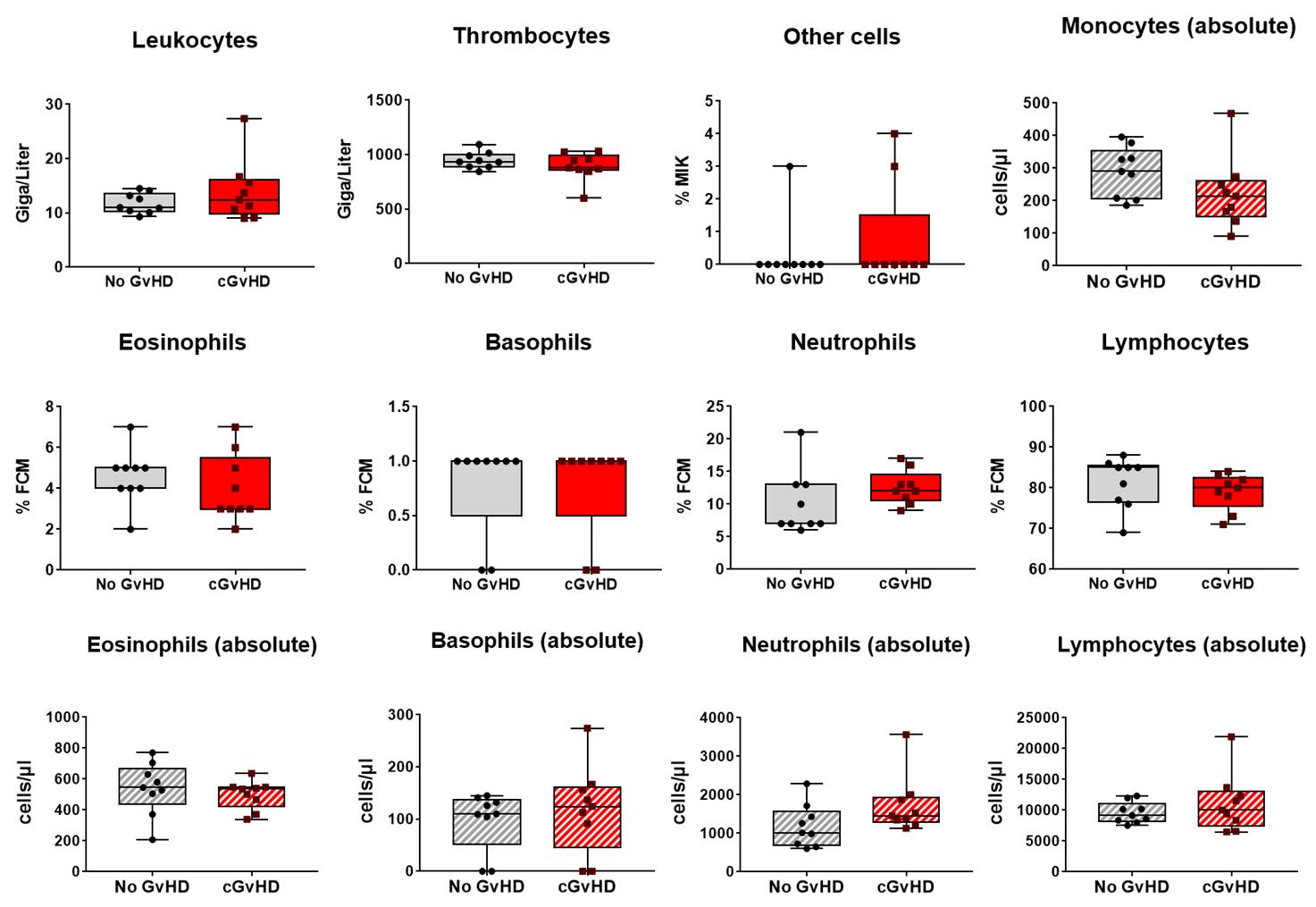


**Supplementary Figure 1: Differential blood count in cGvHD at d+90 after transplantation in C57/BL6🡪BDF.** Blood was sampled from mice and differential blood count was performed by Synlab, Berlin. Data pooled from 2 independent experiments. n=9 per gropu. Error bars represent mean±SEM. *P<0.05, **P<0.01, ***P<0.001, ****P<0.0001 by unpaired students *t*-test.


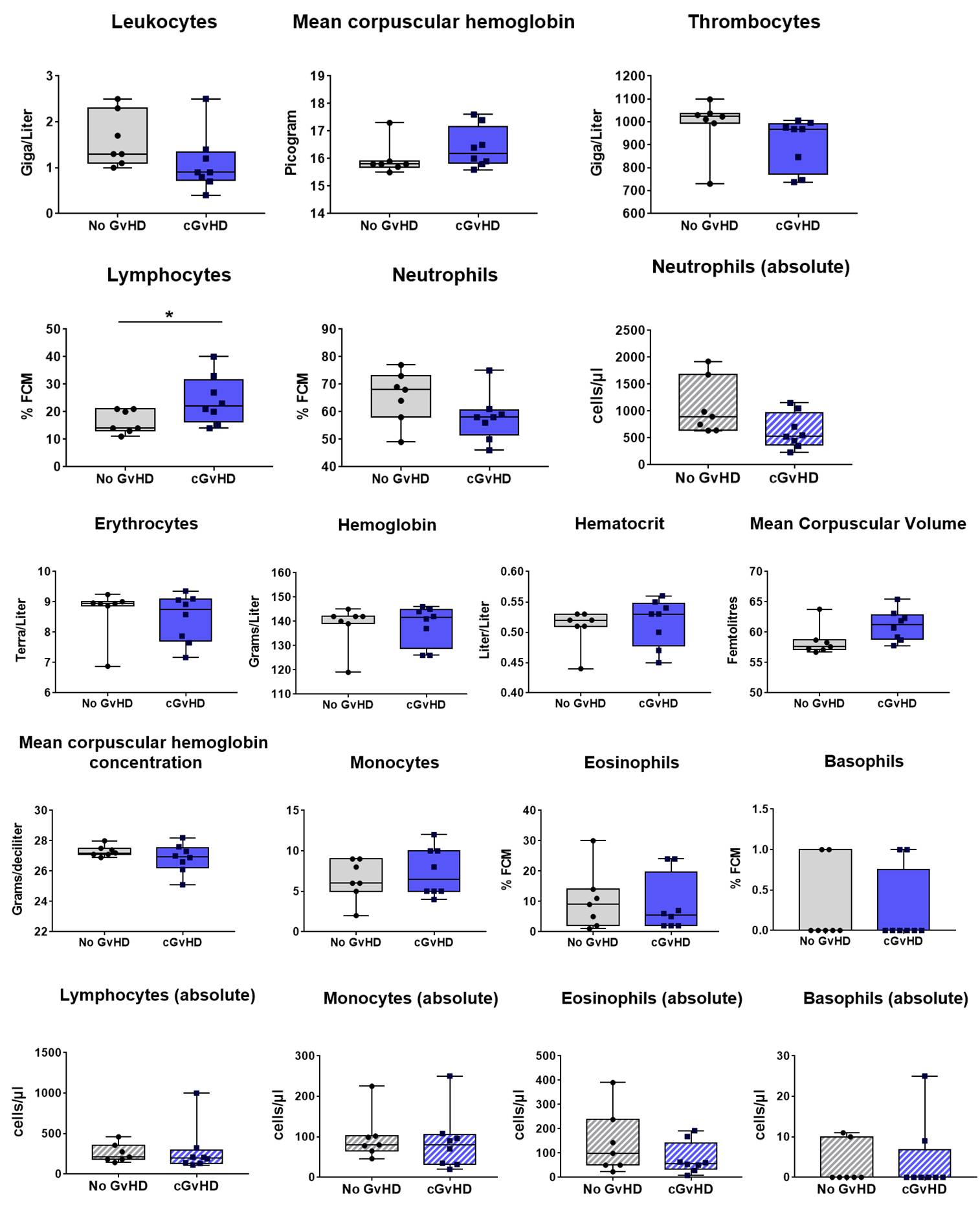


Supplementary Figure 2: Differential blood count in cGvHD at d+90 after xeno-transplantation in huPBMCs🡪NSG. Blood was sampled from mice and differential blood count of murine cells was performed by Synlab, Berlin. Representative data from one out of two experiments. n=8 per group. *P<0.05, **P<0.01, ***P<0.001, ****P<0.0001 by unpaired students *t*-test.
